# Promises and Perspectives of Mesenchymal Stromal Cells in Companion and Farm Animals

**DOI:** 10.3390/ijms27187978

**Published:** 2026-09-08

**Authors:** Thirumala Rao Talluri, Pradeep Nag, Narendra Singh Rathore, Wilfried A. Kues, Dharmendra Kumar, Naresh Selokar, Kamlesh Kumari Bajwa, Mohd Matin Ansari, TK Bhattacharya

**Affiliations:** 1 Equine Production Campus, ICAR-National Research Centre on Equines, Bikaner 334001, India; raotalluri79@gmail.com (T.R.T.);; 2 Southern Regional Station of ICAR-National Dairy Research Institute, Bengaluru 560030, India; drpnbs@gmail.com; 3 College of Veterinary and Animal Sciences, Rajasthan University of Veterinary & Animal Sciences, Bikaner 334001, India; 4 Friedrich Loeffler Institute, Federal Research Institute for Animal Health, Biotechnology/Stem Cell Physiology, 31535 Neustadt, Germany; 5 ICAR-Central Institute for Research on Buffaloes, Hisar 125001, India; 6 ICAR-National Dairy Research Institute, Karnal 132001, India; 7 ICAR-National Research Centre on Camel, Bikaner 334001, India

**Keywords:** mesenchymal stem cell, mesenchymal stromal cell, regenerative medicine, livestock, companion animal, cattle, buffalo, horse, biomolecule

## Abstract

Mesenchymal stromal cells (MSCs) are heterogeneous, multipotent cell populations that have become one of the most extensively investigated cell types in veterinary regenerative medicine. They can be isolated from a wide range of adult and perinatal tissues and have attracted considerable interest because of their immunomodulatory properties, broad secretory activity, and potential to promote tissue repair. However, increasing evidence indicates that MSCs should not be regarded as a uniform therapeutic product, as their biological behaviour is influenced by tissue source, donor characteristics, manufacturing conditions, and the recipient microenvironment. Likewise, their therapeutic effects are now understood to arise predominantly through dynamic interactions with the host, including inflammatory licensing, immunomodulation, secreted bioactive factors, extracellular vesicles, metabolic exchange, and responses to apoptotic cells, rather than through durable engraftment and direct tissue replacement alone. Although clinical evidence supports the use of selected MSC products in canine osteoarthritis and suggests promise for equine musculoskeletal disorders, most other veterinary applications remain preclinical or investigational. This review critically examines current knowledge of MSC biological identity, tissue-source diversity, molecular mechanisms, therapeutic applications, and comparative translational roles in companion and farm animals. It also discusses the challenges that continue to limit clinical translation, including biological heterogeneity, product characterization, potency assessment, manufacturing standardization, and regulatory considerations, and highlights future directions for the development of safe, effective, and evidence-based MSC therapies in veterinary medicine.

## 1. Introduction

Mesenchymal stromal cells (MSCs) have become one of the most widely studied adult cell populations in regenerative medicine. The term mesenchymal stem cell is still used frequently, but mesenchymal stromal cell is more appropriate for heterogeneous populations expanded in culture unless self-renewal and multilineage differentiation have been demonstrated at both clonal and in vivo levels [1]. The minimum criteria proposed by the International Society for Cellular Therapy (ISCT) for human MSCs include plastic adherence, expression of CD73, CD90, and CD105, absence of selected haematopoietic and immune cell markers, and osteogenic, adipogenic, and chondrogenic differentiation in vitro [2]. These criteria remain useful as a reference, but their application to veterinary species is less straightforward because marker expression, antibody availability, and cross-reactivity differ among species. Veterinary MSC identity therefore needs to be established using multiple characteristics [3,4,5].

MSCs have been isolated from bone marrow, adipose tissue, dermis, synovium, reproductive tissues, and several fetal or perinatal sources. Their accessibility, capacity for ex vivo expansion, and broad secretory and immunomodulatory activity have encouraged investigation of both autologous and allogeneic products. Compared with embryonic stem cells and induced pluripotent stem cells, they are generally easier to obtain and expand, involve fewer ethical concerns, and carry a lower risk of teratoma formation [6,7,8]. However, the MSC characteristics vary considerably with species, tissue source, donor characteristics, manufacturing conditions, and recipient environment [6,7,9].

Understanding of MSC biology has evolved considerably in relation to their therapeutic effects. Early work placed considerable emphasis on homing, engraftment, and direct differentiation, although administered MSCs generally showed limited persistence and stable tissue integration [7,10,11]. Current research therefore places greater emphasis on host-directed mechanisms including immunomodulatory, paracrine, and intercellular signalling pathways that contribute to therapeutic responses [10,11,12,13,14,15,16,17,18,19]. These mechanisms and their relevance to veterinary applications are discussed in detail in this review.

Veterinary MSC research has not developed uniformly across species. Much of the clinical literature is dominated by equine and canine studies, particularly for conditions such as osteoarthritis and tendon or ligament injuries [20,21]. Pigs, sheep, and goats have been mainly used as translational models, where human-scale procedures, biomaterial testing, and structural repair have been evaluated [22]. In large ruminants such as cattle and buffalo, research has focused more on reproductive and mammary biology, production-related disorders, developmental studies, and agricultural biotechnology [23,24,25,26]. These differences highlight the need to interpret MSC evidence according to the species, disease context, product characteristics, and intended application.

Wide variation in MSC products and study designs further complicates comparisons across studies, particularly in relation to product characterization, potency, manufacturing, and safety [3,4,27]. Against this background, this review examines the biological identity and molecular actions of MSCs together with the current evidence for their therapeutic applications in companion and farm animals. Particular attention is given to the challenges that influence their translation into veterinary practice. Clinical findings are distinguished from preclinical and mechanistic evidence to provide a balanced view of the current status and future potential of veterinary MSC research.

### Search Strategy and Scope

This narrative review was developed after searching major scientific databases, including PubMed, Scopus, and Google Scholar. Search terms included mesenchymal stem cell, mesenchymal stromal cell, and MSC, combined with relevant animal species and terms related to tissue sources, immunomodulation, molecular mechanisms, extracellular vesicles, regenerative medicine, therapeutic applications, safety, potency, manufacturing, and clinical translation. Peer-reviewed publications in English were considered for inclusion. Veterinary studies were prioritized wherever available, while human and rodent studies were used selectively when direct veterinary evidence for specific molecular mechanisms was limited. Literature available up to August 2026 was considered, with recent publications prioritized while retaining seminal studies that established key concepts. Regulatory and safety-related information was derived primarily from relevant U.S. FDA and European regulatory guidance and standards, with appropriate citations. Clinical, preclinical, and mechanistic evidence was considered separately to minimize overinterpretation of experimental or mechanistic findings as established clinical efficacy.

## 2. Biological Characteristics and Functional Identity of Mesenchymal Stem/Stromal Cells

MSCs comprise heterogeneous, culture-expanded populations isolated from multiple tissues. Their identity cannot be inferred from plastic adherence, surface marker expression, or trilineage differentiation alone, because these properties vary with tissue source, donor, species, and manufacturing conditions. A biologically meaningful definition therefore requires convergent evidence linking cellular phenotype with relevant function [1,3,4,6,7].

### 2.1. Definition and Biological Identity of Mesenchymal Stem/Stromal Cells

Friedenstein and colleagues first described plastic-adherent, colony-forming fibroblastic precursors from bone marrow that generated bone and supportive stroma after transplantation [28]. Caplan later introduced the term mesenchymal stem cells for cells proposed to generate several mesenchymal tissues. These observations established the conceptual basis of MSC research [29]. Subsequent studies, however, showed that conventional culture-expanded preparations are heterogeneous and do not necessarily represent a uniform population of endogenous stem cells [30].

The interpretation of MSC function has changed accordingly. Durable engraftment and direct differentiation may occur in selected experimental settings, but they rarely explain the full therapeutic response. Current evidence indicates that MSC activity depends largely on interactions with host tissues and immune cells rather than on permanent structural replacement [7,10,12]. Caplan proposed the alternative term medicinal signalling cells to emphasize these host-directed effects. This expression is useful conceptually, but it has not replaced the internationally accepted MSC nomenclature [10]. Current guidance recommends retaining the acronym MSC, specifying the tissue of origin and avoiding claims of stemness based solely on culture phenotype [1].

The ISCT minimum criteria for human MSCs include plastic adherence, expression of CD73, CD90, and CD105, absence of selected haematopoietic and immune cell markers, and osteogenic, adipogenic, and chondrogenic differentiation in vitro. These criteria remain a useful reference point, but direct transfer to veterinary species is problematic [2]. Antigen expression differs among species and tissues; validated species-specific antibodies remain unavailable for several domestic animals, and antibodies raised against human proteins may show weak or inconsistent cross-reactivity. Passage, confluence, inflammatory exposure, enzymatic tissue processing, and cryopreservation may further alter the measured phenotype [4,31,32,33].

Veterinary MSC identity should therefore be established using several complementary lines of evidence. Studies should report the species, donor characteristics, tissue and anatomical compartment, isolation method, culture and expansion history, morphology, clonogenicity, immunophenotype, and validation of the antibodies used. Where therapeutic use is proposed, at least one function linked to the intended mechanism of action should also be examined. This multidimensional approach is necessary because populations that satisfy conventional phenotypic criteria may still differ substantially in proliferation, secretory activity, immune regulation, haemocompatibility, and therapeutic potency [4,27].

### 2.2. Tissue Sources and Biological Diversity of MSCs

MSCs or MSC-like stromal populations have been isolated from a wide range of adult, fetal and perinatal tissues. These include bone marrow, adipose tissue, dermis, skeletal muscle, synovium, periosteum, tendon, dental pulp, endometrium, ovary, liver, fetal lung, placenta, umbilical cord, Wharton’s jelly, amniotic membrane, and amniotic fluid. This broad distribution offers practical flexibility, but tissue origin is also a major source of biological variation [6,23,24,25,26,34,35,36].

Comparative transcriptomic and single-cell studies indicate that MSC preparations retain tissue-associated molecular and functional differences and contain multiple subpopulations. Cells obtained from different tissues should therefore not be regarded as interchangeable simply because they express similar markers or undergo trilineage differentiation [34,35]. Source selection should instead be guided by the intended indication, harvesting feasibility, manufacturing requirements, and experimentally demonstrated function. Comparative studies in companion animals likewise show that species and tissue source influence cellular phenotype and biological behaviour [36].

#### 2.2.1. Bone Marrow-Derived MSCs

Bone marrow remains the classical and most extensively characterized source of MSCs and continues to serve as the reference against which many alternative tissue sources are compared. Bone marrow-derived preparations have well-established clonogenic, osteogenic, and chondrogenic properties and have been studied extensively in bone, cartilage, tendon, and ligament repair. Their long history of investigation has also generated considerable information on expansion, safety, and manufacturing [6,29].

Harvesting bone marrow is nevertheless invasive and requires sedation or anaesthesia. Colony-forming stromal progenitors represent only a small proportion of marrow nucleated cells, so substantial in vitro expansion is usually required. Yield and function are affected by donor age and health, skeletal site, aspirate quality, and culture conditions. Bone marrow-derived MSCs have been characterized in cattle, buffalo, pigs, dogs, and horses, while passage-associated reductions in colony formation and proliferative capacity have been reported in veterinary preparations. Bone marrow therefore remains an important biological reference source, but it may be less suitable where minimally invasive collection, high initial cell yield, or rapid manufacturing is required [37,38].

#### 2.2.2. Adipose Tissue-Derived MSCs

Adipose tissue is widely used because it is abundant and generally yields a comparatively large stromal vascular fraction. Collection may be combined with routine surgical procedures, or tissue that would otherwise be discarded may be used, although the invasiveness of harvesting differs by anatomical site and species. Adipose-derived MSCs from major veterinary species commonly show fibroblast-like morphology, expression of conventional mesenchymal markers, and trilineage differentiation [33,39].

These cells often expand efficiently and may offer a practical advantage when clinically relevant doses are needed. They also display immunomodulatory, trophic, and angiogenesis-associated activities. Adipose tissue, however, is not a uniform organ. Subcutaneous, visceral, periarticular, and other depots differ in vascularity, cellular composition, and metabolic exposure [6,39]. A greater initial cell or colony yield does not constitute evidence of superior therapeutic efficacy. Comparative evidence instead supports matching the depot and tissue source to the intended application and verifying the relevant biological function in the final product [34,35].

#### 2.2.3. Perinatal Tissue-Derived MSCs

Perinatal sources include the placenta, umbilical cord and its defined compartments, Wharton’s jelly, amniotic and chorionic membranes, amniotic fluid, and, where successfully isolated, umbilical cord blood. These materials can often be collected at parturition without additional morbidity to the dam or neonate, making them attractive for donor screening and cell banking [40,41,42,43].

Perinatal MSC preparations frequently show rapid proliferation and prolonged expansion compared with adult tissue cells, a feature often attributed to their developmental origin and relatively immature stromal phenotype. This behaviour is not universal, however, and must be established for each species, anatomical compartment, and manufacturing process. MSC-like populations have been isolated from bovine and buffalo umbilical cord, Wharton’s jelly, amnion, and amniotic fluid [40,41,42,43]. Related populations have also been described in canine and equine perinatal tissues [33,39].

These tissues should not be treated as biologically equivalent. Wharton’s jelly, cord lining, perivascular regions, cord blood, amnion, chorion, and placental compartments differ in developmental origin and cellular composition and may therefore yield stromal populations with distinct phenotypes and functions. Placenta, umbilical cord, and fetal membranes collected at birth are generally better described as perinatal or extraembryonic rather than simply fetal. Claims that perinatal MSCs are uniformly more primitive, hypoimmunogenic, or therapeutically superior should be avoided unless supported by direct comparative evidence [40,41,42,43].

#### 2.2.4. Specialized Tissue-Derived Stromal Populations

MSC-like stromal populations have also been isolated from endometrium, ovary, dental pulp, periodontal tissue, dermis, synovium, periosteum, tendon, skeletal muscle, fetal lung, liver, and several other tissues. These sources may retain characteristics associated with their native microenvironment and may therefore offer biological advantages for selected applications [44,45,46,47].

Bovine endometrial stromal populations have shown clonogenicity, mesenchymal-marker expression and mesodermal differentiation, while reproductive stage and uterine disease can alter their properties [44,45]. Ovarian stromal populations have also been investigated in dogs and livestock. Expression of stemness-associated markers or demonstration of in vitro differentiation, however, does not by itself establish an in vivo regenerative function [46].

Dental pulp and periodontal tissues contain stromal populations with developmental links to the neural crest. These cells may display osteogenic, odontogenic and neurotrophic properties, supporting their investigation in dental, craniofacial, and neuroregenerative applications. Single-cell analyses nevertheless demonstrate substantial tissue-, donor-, and subpopulation-level heterogeneity even among closely related dental sources. Veterinary clinical evidence remains limited, and specialized therapeutic claims are best presented as emerging rather than established [47].

Other tissue-associated populations may also possess potentially useful properties, including matrix production by synovial- or tendon-derived stromal cells and reproductive relevance of endometrial populations. The ability to isolate plastic-adherent cells from a particular tissue is not, however, sufficient justification for therapeutic development. Each source should be evaluated for reproducible yield, biological stability, safety, scalability, and indication-relevant potency. The tissue and anatomical compartment should also be incorporated into MSC nomenclature and reported consistently [1,4].

### 2.3. Core Biological Characteristics of MSC Preparations

Under conventional culture conditions, MSC preparations generally adhere to tissue-culture plastic and acquire a spindle-shaped or fibroblast-like morphology. Colony-forming unit-fibroblast assays provide an estimate of clonogenic adherent progenitors, but the result depends strongly on seeding density, medium, incubation period, and scoring method. Neither morphology nor plastic adherence is specific to MSCs, because fibroblasts and other stromal populations can show similar characteristics [2,3,4].

Immunophenotyping is therefore an important component of characterization, although no universal veterinary marker panel has been validated across species, tissues, and laboratories. CD29, CD44, CD73, CD90, CD105, and CD166 are among the markers commonly evaluated as positive markers in veterinary MSC preparations, while haematopoietic and endothelial markers such as CD34, CD45, and CD31 are generally used as exclusion markers. However, expression patterns vary with species and tissue source. CD44 and CD90 are frequently reported across bovine, buffalo, canine, equine, and porcine MSC preparations, whereas CD29, CD73, CD105, CD166, and related markers show greater variability or are not uniformly assessed across species. Antibody clone, species reactivity, fluorochrome, viability discrimination, gating strategy, and appropriate controls should therefore be reported. Absence of a marker does not automatically exclude an MSC preparation when species-specific expression or antibody cross-reactivity has not been established [23,25,31,32,33,36,37,38,48]. Representative immunophenotypic characteristics reported across veterinary species are summarized in Table 1.

Collectively, these studies demonstrate substantial overlap in veterinary MSC immunophenotypes, particularly for CD44 and CD90, but they do not support a universal cross-species marker panel. CD29, CD73, CD105, and CD166 are also frequently reported, although their expression and the availability or cross-reactivity of suitable antibodies vary among species and tissue sources. Likewise, haematopoietic markers are generally absent or expressed at low levels, but individual markers should not be treated as absolute exclusion criteria. Consequently, veterinary MSC characterization should report the species, tissue source, antibody clone and species reactivity, passage, and gating strategy rather than defining MSC identity from a fixed marker combination.

Octamer-binding transcription factor 4 (OCT4), NANOG, SRY-box transcription factor 2 (SOX2), stage-specific embryonic antigens (SSEA), and other pluripotency-associated molecules have been detected in some veterinary MSC cultures. These findings require cautious interpretation. Detection of a transcript or immunoreactive signal does not demonstrate pluripotency and may reflect low-level expression, assay cross-reactivity, pseudogene amplification, or culture-associated changes. Expression of these markers should therefore not be interpreted as evidence of pluripotency without appropriate functional validation [1,2,30,31,32,33].

Osteogenic, adipogenic, and chondrogenic differentiation remains a conventional component of MSC characterization, although it does not independently establish stemness or therapeutic potency [2,30]. Appropriate positive and negative controls should be included, together with complementary histological, biochemical, or molecular assessments where possible [2,6]. Claims of differentiation into neural, hepatic, pancreatic, endothelial, germ cell, or cardiomyocyte-like phenotypes require greater caution because morphological changes or expression of lineage-associated markers alone do not demonstrate acquisition of a mature, functional phenotype [6,30]. Such outcomes are better described as induced or lineage-associated phenotypes unless supported by appropriate functional evidence [1,6,30].

Culture-expanded MSCs may initially show substantial proliferative and clonogenic capacity, but both can decline with prolonged expansion. Passage number alone is an imprecise measure of culture history because laboratories differ in split ratio, seeding density, harvest confluence, and time in culture. Cumulative population doublings, population-doubling time, seeding density, and harvest confluence therefore provide a more informative description of expansion history [27,49].

Low basal expression of major histocompatibility complex (MHC) class II and co-stimulatory molecules has encouraged the development of allogeneic products, but MSCs are not universally immune privileged. Immune recognition varies with species, donor–recipient compatibility, tissue source, inflammatory licensing, culture conditions, and repeat exposure [9]. In horses, repeated intra-articular administration of some allogeneic preparations has produced greater inflammatory responses than autologous treatment, supporting the concept of context-dependent immunogenicity, rather than universal non-immunogenicity [50].

MSCs can respond to chemokines, adhesion molecules, and extracellular matrix signals and may migrate towards injured or inflamed tissues. Systemic delivery, however, often results in early pulmonary sequestration and limited retention within the intended target tissue. Homing is better regarded as a route- and context-dependent process rather than evidence of durable engraftment [7,11]. Therapeutic activity may instead arise from transient interactions with host cells and from recipient responses to cleared or apoptotic MSCs, supporting a shift away from a simple cell-replacement model [14,15,16].

### 2.4. Biological Heterogeneity and the Influence of Manufacturing

MSC heterogeneity arises at several levels, including species, tissue and anatomical compartment, donor, clonal composition, isolation method, culture environment, expansion history, storage, and formulation. These variables can alter proliferation, differentiation, metabolism, secretome composition, immune regulation, and safety. An MSC product is therefore defined not only by its tissue of origin but also by the process used to manufacture it [6,27,34,35].

Donor age and health are important but incompletely standardized sources of variation. Ageing is generally associated with reduced clonogenicity, altered differentiation, oxidative stress, and senescence, although the magnitude of these effects varies among species and tissue sources. In bovine endometrial MSCs, uterine inflammatory disease altered proliferative and transcriptomic properties. Breed, sex, reproductive stage, metabolic status, medication, and concurrent disease may also influence cell behaviour and should therefore be reported [6,27,34,35,45].

Culture medium and supplementation strongly affect MSC phenotype and function. Fetal bovine serum remains widely used in veterinary MSC culture but is chemically undefined, batch variable, and associated with xenogeneic protein, biosafety, and reproducibility concerns. Serum-free, xeno-free, platelet-derived, and species-adapted supplements may improve standardization, although changes in medium composition can also alter cell growth and functional potency. Culture systems should therefore be qualified for the intended species, tissue source, and clinical application [6,27,40,51].

Oxygen tension is another important culture variable because atmospheric oxygen exceeds that of many native stromal niches. Hypoxic culture can modify proliferation, metabolism, migration, differentiation, and secretion, but the response depends on oxygen concentration, exposure duration, passage, tissue source, and species. In buffalo bone marrow-derived MSCs, culture at 5% oxygen was associated with improved colony formation and migration and with HIF–TWIST-related changes. These findings support controlled evaluation of oxygen tension but do not establish a universally optimal concentration [6,27,34,52].

Prolonged expansion can lead to replicative senescence, enlarged and flattened morphology, reduced colony formation, mitochondrial stress, and altered immunological or secretory activity. Quality assessment should therefore include growth kinetics and, where appropriate, measures of senescence and genomic stability. The ability to maintain cells through a high passage number does not by itself demonstrate preservation of product quality [6,27,30,49].

Cryopreservation facilitates banking, transport, and off-the-shelf use, but its effects are product- and protocol-specific. Conventional morphology and marker expression may remain intact while viability, adhesion, metabolic recovery, inflammatory responsiveness, or immunomodulatory function are altered [53,54]. Conversely, optimized protocols can recover functional cells from several tissues. Fresh and cryopreserved products should therefore not be assumed to be equivalent or non-equivalent without direct testing. Post-thaw evaluation should include viability, cellular recovery and indication-relevant functional potency at the intended time of administration [55].

### 2.5. From Phenotypic Characterization to Functional Potency

Plastic adherence, surface markers and trilineage differentiation support MSC identity and comparability but do not measure the biological functions expected to produce clinical benefit. Preparations with similar conventional phenotypes may differ substantially in cytokine responsiveness, immune cell regulation, trophic factor release, extracellular matrix production, angiogenic activity, EV output, and metabolic fitness [1,7].

Potency testing should therefore be linked to the intended mechanism and indication. Products developed for immune-mediated disease may require assays of inflammatory licensing, lymphocyte proliferation, monocyte or macrophage responses, or relevant soluble mediators. Musculoskeletal products may require matrix-related, trophic, anti-inflammatory, angiogenic, or cytoprotective assays, while secretome and EV products require additional assessment of identity, quantity, purity, and biologically relevant activity [18,19,56].

No single assay is likely to capture the activity of a heterogeneous MSC product. A qualified matrix of complementary analytical and functional assays is more defensible when each component is linked to product performance. Potency should be interpreted together with sterility, endotoxin, mycoplasma, viable dose, identity, purity, genomic stability, and batch consistency [57,58].

A veterinary MSC product is consequently defined by the interaction of species, tissue and anatomical compartment, donor, manufacturing process, storage, and intended use. Establishing fitness for purpose requires species-appropriate reagents, transparent expansion and storage data, and one or more indication-relevant functional assays, thereby linking biological identity to therapeutic activity rather than relying on conventional marker panels alone [1,4,27,56,57,58].

## 3. Molecular Mechanisms Underpinning MSC Therapeutic Function

The therapeutic activity of MSCs was initially attributed mainly to homing, durable engraftment, and differentiation into tissue-specific cells. However, biodistribution studies have shown limited long-term persistence after systemic administration and restricted integration in many local applications, indicating that stable cell replacement alone cannot explain the range of functional responses observed after treatment [6,7,10,11,29].

**Figure 1:**
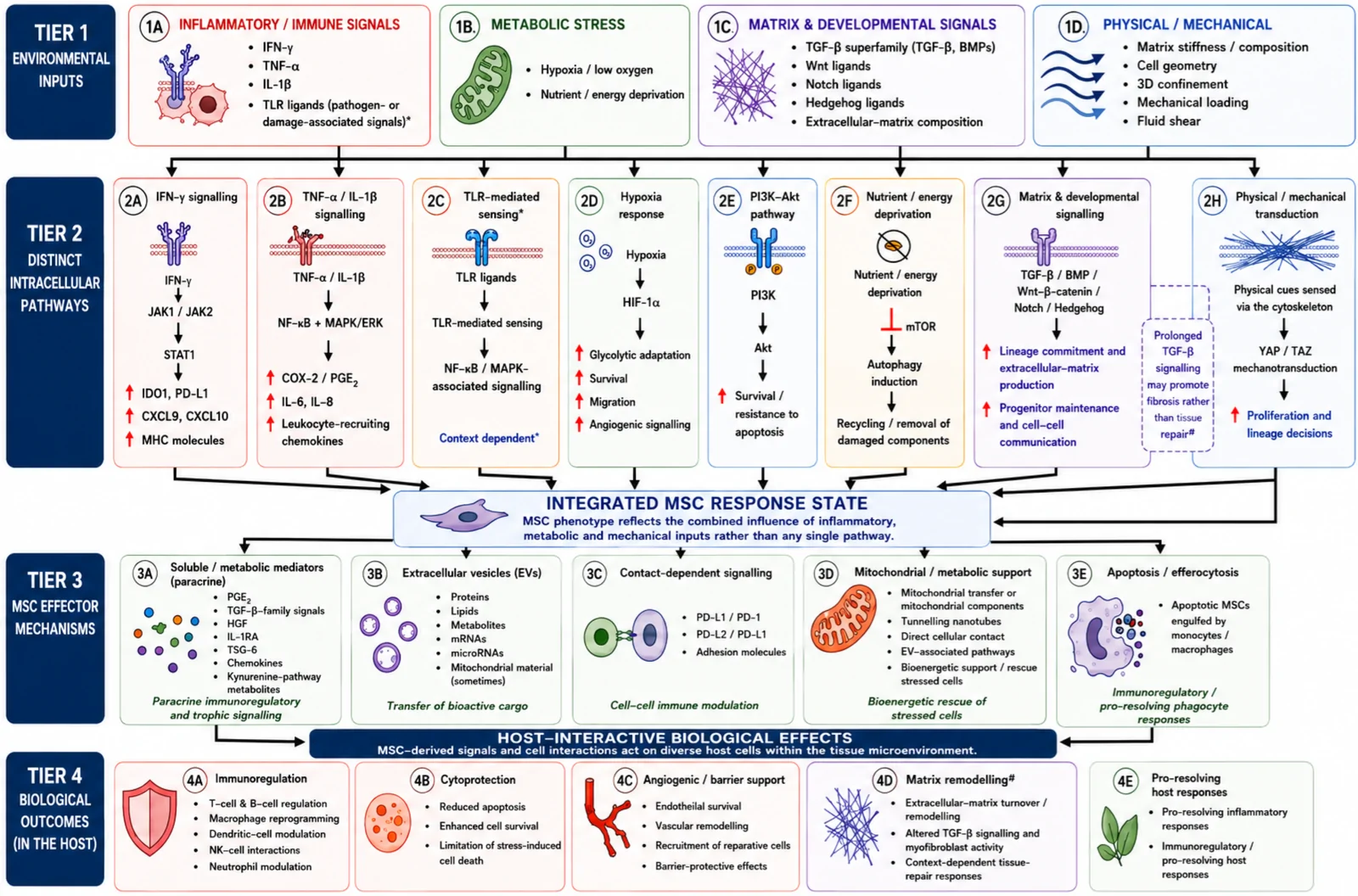
Integrated model of environmental sensing, intracellular signalling, and host-interactive biological effects in MSCs. Environmental inputs, including inflammatory and immune signals, hypoxia, nutrient and energy deprivation, matrix/developmental signals, and physical or mechanical cues, activate interconnected pathways, including JAK–STAT, NF-κB/MAPK, TLR-associated signalling, HIF-1α, PI3K–Akt, mTOR/autophagy, TGF-β–SMAD, BMP, Wnt/β-catenin, Notch, Hedgehog, and YAP/TAZ-associated mechanotransduction. These signals converge to shape an integrated MSC response state and downstream effector mechanisms involving soluble and metabolic mediators, extracellular vesicles, contact-dependent signalling, mitochondrial transfer or metabolic support, and host responses to apoptotic MSCs. Through these mechanisms, MSCs may contribute to immunoregulation, cytoprotection, angiogenic and barrier support, matrix remodelling, and pro-resolving host responses. Solid black arrows indicate pathway activation or downstream signalling, bar-ended lines indicate inhibition, and red upward arrows indicate increased expression or biological activity. * TLR responses are context-dependent and vary with ligand purity, concentration, exposure duration, tissue source, and surrounding cytokines. # Matrix/developmental responses are context-dependent; prolonged TGF-β signalling may promote fibrosis rather than tissue repair. Pathway contributions also vary with species, donor, tissue source, manufacturing process, route of administration, and disease microenvironment. This figure was generated using ChatGPT (OpenAI, GPT-5.6 Sol) based on a conceptual framework conceived by the authors; the scientific content, pathway relationships, labels, and final presentation were reviewed and edited by the authors. Abbreviations: IFN-γ, interferon-gamma; TNF-α, tumour necrosis factor-alpha; IL, interleukin; TLR, Toll-like receptor; JAK, Janus kinase; STAT, signal transducer and activator of transcription; NF-κB, nuclear factor kappa B; MAPK, mitogen-activated protein kinase; ERK, extracellular signal-regulated kinase; PI3K, phosphatidylinositol 3-kinase; Akt, protein kinase B; HIF-1α, hypoxia-inducible factor-1 alpha; mTOR, mechanistic target of rapamycin; IDO1, indoleamine 2,3-dioxygenase 1; PGE_2_, prostaglandin E_2_; TGF-β, transforming growth factor-beta; BMP, bone morphogenetic protein; TSG-6, tumour necrosis factor-stimulated gene-6; HGF, hepatocyte growth factor; IL-1RA, interleukin-1 receptor antagonist; EVs, extracellular vesicles; PD-L1, programmed death-ligand 1; PD-L2, programmed death-ligand 2; PD-1, programmed cell death protein 1; YAP, Yes-associated protein; TAZ, transcriptional co-activator with PDZ-binding motif.

Current evidence instead supports a host-interactive model in which MSCs respond to inflammatory, metabolic, and mechanical signals and influence recipient tissues through soluble mediators, extracellular vesicles, direct cell contact, metabolic exchange, and host responses to short-lived or apoptotic cells [12,14,15,16,17,18,19]. These mechanisms are interconnected and vary according to the MSC product, route of administration, and disease environment. Limited persistence of administered MSCs in an equine model of post-breeding endometritis further supports an important paracrine or host-mediated contribution [59]. These interconnected mechanisms are summarized in Figure 1.

### 3.1. Inflammatory Licensing and Functional Activation

MSCs are not constitutively immunosuppressive to the same degree. Their functional phenotype changes in response to the inflammatory environment through a process commonly described as licensing, priming, or preconditioning. These terms encompass different stimuli, concentrations, and exposure schedules and should therefore be defined experimentally [7,12,13].

Inflammatory cytokines released following tissue injury, particularly interferon-γ (IFN-γ), tumour necrosis factor-α (TNF-α), and interleukin-1β (IL-1β), induce transcriptional and metabolic programmes that alter chemokine secretion, adhesion molecules, antigen-presentation machinery, and immunoregulatory activity [12,13,60]. IFN-γ receptor signalling activates JAK1/JAK2–STAT1 and can induce IDO1, programmed death ligand-1 (PD-L1), CXCL9, CXCL10, and major histocompatibility complex molecules [13,60,61]. TNF-α and IL-1β activate NF-κB and mitogen-activated protein kinase pathways, increasing cyclooxygenase-2-dependent prostaglandin E_2_ (PGE_2_), IL-6, IL-8, and leukocyte-recruiting chemokines [7,12].

Combined IFN-γ and TNF-α exposure often generates a stronger immunoregulatory phenotype than either cytokine alone. Stronger licensing may, however, occur alongside increased expression of MHC molecules and other immunologically recognizable features. Human leukocyte antigen-G (HLA-G)–mediated activity has been described in human MSC systems, but this mechanism should not be transferred directly to veterinary species because HLA molecules are human leukocyte antigens and their functional counterparts differ among animals [7,9,13].

Licensing is a graded and product-dependent response rather than a binary switch. Its magnitude depends on cytokine identity, concentration and exposure time, together with donor, tissue source, passage, differentiation state and baseline potency [7,27,34,35]. Increasing the priming dose also cannot be assumed to convert a weak donor into a therapeutically potent product [60].

Toll-like receptors provide another route through which MSCs detect pathogen-associated and damage-associated signals. Short-term Toll-like receptor 3 (TLR3) and Toll-like receptor 4 (TLR4) stimulation was originally proposed to generate relatively immunosuppressive “MSC2” and pro-inflammatory “MSC1” states, respectively. Subsequent work has shown that these responses vary with ligand purity, concentration, exposure duration, tissue source, and surrounding cytokines [62]. The MSC1/MSC2 framework is therefore more useful as a conceptual model rather than as a fixed biological classification [7,12,62].

Physical signals also shape MSC activation. Mechanical loading, fluid shear, substrate stiffness, cell geometry, and three-dimensional architecture influence inflammatory and trophic signalling. Licensing is better regarded as an integrated response to soluble and physical cues rather than a process driven exclusively by cytokines [63,64].

Veterinary studies confirm both the existence and variability of licensing responses. In porcine bone marrow-derived MSCs, IFN-γ increased IDO1 expression and activity, whereas previous adipogenic or osteogenic differentiation reduced immunomodulatory and migratory properties that were not fully restored by licensing [65]. Canine adipose- and bone marrow-derived MSCs suppress activated T cells through overlapping but tissue-dependent mechanisms involving IDO1 and PGE_2_ [66]. IFN-γ stimulation can also enhance IDO–kynurenine pathway activity in canine MSCs [67]. In equine MSCs, inflammatory or TLR3 priming can increase selected immunoregulatory functions, although responses differ among donors, tissue sources, and experimental conditions [68,69]. These findings support species- and product-specific optimization rather than direct transfer of human licensing protocols to veterinary MSC preparations [70].

### 3.2. Immunomodulatory Mechanisms

MSC immunomodulation involves adaptive and innate immune cells and is mediated through soluble factors, metabolites, extracellular vesicles (EVs), adhesion-dependent contact, and host-mediated processing of administered cells [12,14,15,16]. MSCs should not be described as globally immunosuppressive, as their effects may be suppressive, supportive or pro-resolving depending on the activation state of the immune system and the surrounding signals [7,12].

#### 3.2.1. Regulation of T and B Lymphocytes

Activated T lymphocytes are among the best-characterized targets of MSC-mediated regulation. IFN-γ-induced IDO1 catabolizes tryptophan into kynurenine pathway metabolites, restricting a substrate required for T-cell proliferation while generating metabolites that influence T-cell survival and differentiation [61]. PGE_2_, transforming growth factor-β1 (TGF-β1), PD-L1/programmed death ligand-2 (PD-L2), galectins, and other mediators can further reduce T-cell proliferation, inflammatory cytokine production, and cytotoxic activity [7,12,60,66].

Species differences are mechanistically important. Human MSCs commonly use IFN-γ-inducible IDO1 as a major immunoregulatory mechanism, whereas murine MSCs rely more strongly on inducible nitric oxide synthase and nitric oxide under comparable inflammatory conditions [71]. Canine MSCs appear to use IDO1 and PGE_2_ to different extents depending on tissue source and stimulation, indicating that veterinary species cannot be assumed to follow either the human or murine pattern exactly [66,67].

Contact-dependent pathways complement soluble and metabolic mechanisms. PD-L1 and PD-L2 interact with PD-1 on activated T cells to attenuate downstream signalling, while adhesion molecules maintain close proximity between lymphocytes and MSC-derived immunoregulatory mediators. These pathways act together rather than as mutually exclusive mechanisms [60,66].

MSCs can favour the induction or expansion of regulatory T-cell populations through combinations of TGF-β1, PGE_2_, and contact-mediated signalling. The magnitude of regulatory T-cell induction varies with MSC source, inflammatory activation, immune cell composition, and assay design and should therefore be demonstrated experimentally rather than assumed for every product [61].

Although T-cell regulation has been studied most extensively, MSCs also influence humoral immunity. Human MSCs can inhibit B-cell proliferation, differentiation and immunoglobulin production under some activation conditions, whereas other studies have reported B-cell expansion or plasma cell differentiation depending on the B-cell subset, activation stimulus, culture conditions and MSC-to-B-cell ratio [72,73]. Comparable mechanisms remain insufficiently characterized in veterinary species, so MSC effects on B cells should be regarded as context-dependent rather than uniformly suppressive [3,4,5,20,23,24,25]. C-C motif chemokine ligand 2 (CCL2)/monocyte chemoattractant protein-1 (MCP-1) has also been implicated in MSC-mediated regulation of B-cell proliferation and differentiation, although direct evidence for this mechanism remains limited in veterinary species [74].

#### 3.2.2. Macrophage Reprogramming

Beyond adaptive immunity, macrophages are now considered major intermediaries of MSC therapeutic activity. MSC-derived PGE_2_ can act on host macrophages to increase IL-10 production, which is more precise than attributing all measured IL-10 directly to MSC secretion [75]. Tumour necrosis factor-stimulated gene-6 (TSG-6), TGF-β-related signals, EVs, and metabolic interactions may further reduce inflammatory macrophage programmes and promote phagocytic, tissue-remodelling, and pro-resolving functions [12,15,76]. MSC-derived CCL2/MCP-1 can also contribute to monocyte/macrophage crosstalk by recruiting C-C chemokine receptor type 2 (CCR2) positive monocytes, which may subsequently contribute to immunoregulatory macrophage responses [77].

The common description of an M1-to-M2 switch is useful for simplified reporting but does not adequately represent the multidimensional and tissue-specific macrophage states observed in vivo. Macrophage responses are therefore better assessed using functional endpoints such as cytokine production, metabolism, phagocytosis, oxidative activity, and tissue-remodelling behaviour [75,76,78].

Macrophage reprogramming may persist after viable MSCs are no longer detectable. In a chronic intestinal inflammation model, early PGE_2_-dependent suppression was followed by sustained anti-inflammatory macrophage programming associated with efferocytosis of short-lived MSCs. This sequential response may help explain prolonged therapeutic effects after transient MSC exposure, although its relevance requires validation in each disease and species [78].

#### 3.2.3. Dendritic Cells, Natural Killer Cells, and Neutrophils

MSCs can interfere with dendritic cell differentiation, maturation, co-stimulatory molecule expression, and antigen-presenting function [79]. Canine MSCs also suppress dendritic cell activation through several biochemical pathways, providing direct evidence that this interaction extends beyond human systems. The effect depends on dendritic cell maturation stage and inflammatory environment and should not be interpreted as complete suppression of antigen presentation [66].

The interaction between MSCs and natural killer (NK) cells is reciprocal. Human MSCs can reduce NK cell proliferation, cytokine production and cytotoxicity through IDO1, PGE_2_ and contact-dependent mechanisms, whereas activated NK cells can recognize and induce MSC death. This bidirectional interaction may contribute to apoptosis-dependent immunomodulation, although direct veterinary evidence remains limited [80,81].

MSC effects on neutrophils are similarly context-dependent. Human MSCs can prolong neutrophil survival through IL-6-dependent signalling and may support antimicrobial activity through factors such as LL-37. Because comparable mechanisms remain poorly characterized in veterinary species, MSC treatment in infectious disease should be described cautiously as immunoregulatory rather than uniformly immunosuppressive or antimicrobial [82,83].

### 3.3. Secretome-Mediated Tissue Protection and Repair

The MSC secretome comprises soluble proteins, lipid mediators, metabolites, extracellular matrix components, and membrane-enclosed EVs released constitutively or after environmental stimulation [12,18]. Its composition changes with tissue source, donor, species, passage, oxygen tension, inflammatory licensing, culture medium, three-dimensional organization, and cellular stress. Secretome-based mechanisms provide a plausible explanation for functional improvements despite limited MSC persistence, but a compositional profile alone does not demonstrate causality in vivo [18,34,35,51,52].

#### 3.3.1. Anti-Inflammatory and Immunoregulatory Programmes

PGE_2_, TGF-β-family signals, hepatocyte growth factor, IL-1 receptor antagonist, TSG-6, chemokines, and kynurenine pathway metabolites contribute to the immunoregulatory activity of MSC preparations [12,61,75,76,84].

TSG-6 can reduce inflammatory leukocyte recruitment and signalling [76], while MSC-derived IL-1 receptor antagonist has been associated experimentally with attenuation of inflammatory and fibrotic injury. No single mediator is sufficient to explain the activity of every MSC preparation. Mechanistic analyses should therefore combine pathway perturbation with multiplex molecular and functional measurements rather than infer potency from the concentration of one molecule [84].

#### 3.3.2. Angiogenic and Barrier-Protective Programmes

MSCs can release vascular endothelial growth factor (VEGF), angiopoietin-1, fibroblast growth factors, placental growth factor, hepatocyte growth factor, and chemokines that support endothelial survival, vascular remodelling, and recruitment of reparative host cells [12,85]. Conditioned medium from MSCs has stimulated collateral vessel formation through a coordinated arteriogenic cytokine response, supporting a paracrine basis for vascular effects [85].

Keratinocyte growth factor and angiopoietin-1 have been implicated in epithelial-barrier protection in experimental injury models, while matrix metalloproteinases participate in extracellular matrix turnover, cell migration, and vascular remodelling. These findings do not establish that every MSC product is uniformly pro-angiogenic. Excessive or poorly controlled angiogenesis may be undesirable in neoplastic, retinal, or fibrotic disease, and the response depends on recipient tissue and disease stage [12].

#### 3.3.3. Cytoprotection, Matrix Remodelling, and Antifibrotic Activity

MSC-derived growth factors, lipid mediators and EV cargo can limit stress-induced cell death and modify oxidative and inflammatory signalling. In acute kidney injury models, MSC-derived microvesicles protected renal tubular cells through transferred RNA and protein cargo, supporting a paracrine mechanism that does not require durable donor cell integration [18,86].

MSCs may also influence extracellular matrix turnover by altering inflammatory cell recruitment, TGF-β signalling, myofibroblast activation, and the balance between matrix metalloproteinases and their inhibitors. These effects are context-dependent: signals that support early repair may promote fibrosis when they are excessive or prolonged. The MSC secretome should therefore be viewed as an indication-specific biological programme rather than as inherently antifibrotic, and potency assays should be matched to the therapeutic function being evaluated [12,56,57,58,84].

### 3.4. Extracellular Vesicles and RNA-Mediated Communication

Extracellular vesicles (EVs) contribute to MSC paracrine activity by transferring proteins, lipids, metabolites, messenger RNAs, microRNAs, and, in some preparations, mitochondrial material to recipient cells. MSC-derived EVs are being investigated as cell-free therapeutics because they can reproduce selected biological effects of parental cells while avoiding replication, differentiation and some physical risks associated with viable cell administration [18,19,86].

EVs are not equivalent to the complete MSC response. They cannot adapt dynamically to the recipient after administration, and their cargo varies with tissue source, donor, passage, culture medium, oxygen tension, inflammatory priming, and cellular stress. EV-associated microRNAs may influence inflammatory signalling, apoptosis, angiogenesis, fibrosis, and macrophage function, but attribution to an individual RNA requires functional validation rather than detection alone [18,19].

The term exosome should be used only when endosomal biogenesis has been demonstrated; otherwise, operational terms such as small or medium/large extracellular vesicles are preferable. The 2023 Minimal Information for Studies of Extracellular Vesicles (MISEV2023) guidelines recommend transparent reporting of producer cells, culture conditions, separation procedures, contaminants, storage, dose definition, and functional testing [19]. EV products also require control of purity, cargo stability, batch consistency, biodistribution, and indication-specific potency and should not be assumed to be safer or more effective than parental MSCs without direct comparative evidence [18,19,56].

### 3.5. Mitochondrial Transfer and Metabolic Rescue

MSCs can transfer mitochondria or mitochondrial components to injured cells through tunnelling nanotubes, direct cellular contacts, and EV-associated pathways in selected experimental systems [17,87,88,89,90]. The trafficking GTPase Miro1 contributes to mitochondrial movement along the cytoskeleton and can influence the efficiency of intercellular transfer [87]. In lung injury models, transfer of functional mitochondria from MSCs to epithelial cells improved bioenergetic function, while transfer to macrophages enhanced oxidative phosphorylation and phagocytic activity [88,89,90].

Evidence of mitochondrial DNA or mitochondrial proteins within an EV fraction does not, however, establish delivery of intact, respiration-competent organelles. Direct transfer of functional mitochondria should therefore be distinguished from transfer of mitochondrial fragments, nucleic acids, or signals that stimulate endogenous mitochondrial biogenesis [19,90].

Most detailed evidence derives from human cells and rodent models. Direct veterinary evidence remains limited despite the relevance of mitochondrial dysfunction to equine musculoskeletal injury, canine neurological disease, porcine cardiovascular models, and livestock reproductive disorders. Mitochondrial transfer should therefore be regarded as an emerging and biologically plausible mechanism rather than an established basis for routine veterinary MSC therapy [17,87,88,89,90].

### 3.6. Apoptosis, Efferocytosis and Host-Mediated Immunomodulation

A major change in the MSC paradigm is the recognition that apoptosis of administered cells may contribute to therapeutic activity rather than simply representing treatment failure [14,15,16]. Following intravenous delivery, many MSCs are retained within the pulmonary microvasculature and cleared within a relatively short period [11,14,15,16]. Cytotoxic T cells and NK cells can induce MSC apoptosis, while mechanical stress, complement activation, and exposure to an inhospitable environment may also reduce survival [14,81].

Apoptotic MSCs are engulfed by monocytes and macrophages through efferocytosis [14,15,16]. This uptake can induce immunoregulatory and pro-resolving programmes involving IDO1, kynurenine metabolism, PGE_2_, and IL-10-associated responses in recipient phagocytes [14,15,16,91]. Experimental impairment of MSC apoptosis reduced therapeutic efficacy in the models examined by Pang and colleagues, supporting apoptosis as a mechanistic component rather than merely a marker of product failure [16].

Viable cell signalling and apoptotic cell clearance may occur sequentially rather than as competing explanations [15,78,91]. Human monocytes that engulfed physiologically active MSCs developed different and stronger immunoregulatory features than those exposed to heat-inactivated cells, indicating that the pre-apoptotic state of the MSC influences the subsequent phagocyte response [91]. Pre-generated cryopreserved apoptotic MSC preparations have retained biological activity in experimental testing, suggesting a possible product development strategy, although this approach remains investigational [92].

Recipient biology and administration route may both influence apoptosis-dependent MSC activity. Cytotoxic cell function and phagocytic clearance can affect how a recipient responds to the same product, suggesting that potency assessment may need to consider both MSC susceptibility to appropriate death signals and the subsequent response of recipient-like phagocytes. Route-specific differences are equally important: intravenous delivery favours pulmonary sequestration and systemic clearance, whereas local administration may expose MSCs to distinct survival, death and efferocytosis pathways. Findings from systemic delivery models should therefore not be generalized automatically to intra-articular, intralesional or scaffold-based therapies [13,14,15,16].

### 3.7. Integrated Signalling Networks Governing MSC Therapeutic Activity

MSC behaviour is governed by interconnected signalling networks that integrate cytokines, nutrients, oxygen availability, extracellular matrix composition, and mechanical forces. Conservation of a pathway across mammals does not establish identical quantitative behaviour in humans, mice, dogs, horses, pigs, cattle, or buffalo. Veterinary studies should therefore state clearly whether a pathway was measured directly in the target species or inferred from broader mammalian evidence [6,12,52,63,64].

#### 3.7.1. Inflammatory and Immunoregulatory Signalling

MSCs are exposed to multiple inflammatory signals simultaneously rather than to individual cytokines in isolation. IFN-γ, TNF-α, IL-1β, and TLR-mediated signals activate overlapping JAK–STAT, NF-κB, and MAPK pathways, including extracellular signal-regulated kinase (ERK) signalling, and the resulting crosstalk determines the strength and character of the MSC response. Depending on the combination and duration of these signals, MSCs may increase immunoregulatory mediators, chemokines, and adhesion molecules while also modifying their survival, migration and stress responses [6,7,12,13,60,61,62]. Thus, the inflammatory phenotype of an MSC is better understood as the integrated outcome of several signalling inputs rather than the activity of a single pathway.

Direct veterinary studies have demonstrated inducible IDO-related and inflammatory-response pathways in canine, equine, and porcine MSCs, but the magnitude and functional consequence differ among tissue sources and donors [65,66,67,68,69,70].

#### 3.7.2. Survival, Autophagy, Immunometabolism, and Hypoxic Adaptation

MSCs, upon administration, may encounter various stressors such as hypoxia and nutrient deprivation. Under such conditions, several stress-responsive pathways are engaged, including hypoxia-inducible factor-1α (HIF-1α), phosphoinositide 3-kinase–Akt (PI3K–Akt) signalling, and mechanistic target of rapamycin (mTOR)–regulated autophagy. HIF-1α facilitates adaptation to a low-oxygen environment by altering glycolytic metabolism, survival, migration, and angiogenic signalling [52]. PI3K–Akt signalling supports MSC survival and resistance to apoptosis [6,93]. Nutrient and energy deprivation can suppress mTOR activity, thereby promoting autophagy, which facilitates recycling of cellular components and removal of damaged proteins and organelles during metabolic stress [6,49].

These stress-adaptive responses are accompanied by metabolic reprogramming, and the balance between glycolysis, mitochondrial respiration, and redox state can influence MSC secretory and immunoregulatory functions [12]. However, when metabolic stress is prolonged or exceeds the adaptive capacity of the cells, mitochondrial dysfunction and cellular damage may accumulate, favouring senescence, reduced proliferation, and altered secretory activity [49].

In buffalo bone marrow-derived MSCs, culture at 5% oxygen improved colony formation, migration, polarity, and karyotypic stability in association with HIF–TWIST signalling [52]. This provides direct veterinary evidence that reduced oxygen tension can modify MSC properties under culture conditions but does not establish the degree of hypoxia experienced by MSCs in vivo or a universally optimal oxygen concentration.

#### 3.7.3. Differentiation, Matrix Remodelling, and Mechanotransduction

MSC differentiation and matrix-remodelling responses are shaped by biochemical and physical signals acting together. TGF-β–SMAD, bone morphogenetic protein (BMP), and Wnt/β-catenin signalling influence lineage commitment and extracellular matrix production, while Notch and Hedgehog contribute to progenitor maintenance and cell–cell communication. The outcome of these signals depends on their strength and duration as well as the surrounding cellular environment; for example, prolonged TGF-β signalling may promote fibrosis rather than tissue repair [6,30].

At the same time, MSCs sense the physical properties of their environment, including matrix stiffness, cell geometry, three-dimensional confinement, and mechanical loading. These cues are transmitted through the cytoskeleton and mechanotransducers such as Yes-associated protein (YAP) and transcriptional co-activator with PDZ-binding motif (TAZ), thereby influencing proliferation and lineage decisions [63,64]. Thus, differentiation is determined not only by soluble signalling molecules but also by the physical environment in which MSCs receive those signals.

In vivo, MSCs encounter these signals simultaneously. Within an injured or remodelling tissue, biochemical signals act alongside changes in oxygen and nutrient availability, matrix composition, stiffness, and mechanical forces. The resulting MSC phenotype therefore reflects the combined influence of inflammatory, metabolic, and mechanical inputs rather than any single signalling pathway [6,12,52,63,64]. This integrated cellular state can influence whether MSCs maintain survival, alter their secretory activity, undergo lineage-associated differentiation or participate in tissue remodelling. Consequently, therapeutic potency cannot be predicted reliably from a single pathway, surface marker, or secreted factor [56,57,58].

### 3.8. Molecular Basis of Variable Therapeutic Responses

The diversity of MSC mechanisms helps explain variable outcomes among products and indications. A preparation selected for T-cell suppression may not be optimal for cartilage matrix support, angiogenesis, or antimicrobial activity, while cells with robust osteogenic differentiation may not produce the most appropriate secretome for immune-mediated disease [6,7,56,57,58]. MSC potency should therefore be considered in relation to the intended therapeutic function rather than as a single universal property.

The response also depends on the recipient environment. The same MSC preparation may behave differently depending on the local inflammatory and metabolic conditions, immune cell activity, and tissue environment encountered after administration. Route of delivery further modifies these interactions: intravenous administration favours pulmonary and systemic host cell interactions, whereas local delivery alters cell retention, matrix contact, and the populations of recipient cells exposed to the product [14,15,16,52,63,64]. Thus, therapeutic activity emerges from the interaction between the characteristics of the MSC product and the biological environment of the recipient.

Mechanistic claims should distinguish direct evidence in the target species from extrapolation, and potency assays should be linked to the intended indication rather than treated as universal release tests [56,57,58]. Recent canine and equine studies also demonstrate that priming responses vary with donor, dose, and stimulus [69,70]. Linking these measurable product responses with therapeutic outcomes will therefore be important for developing indication-specific potency assays and improving consistency between MSC products.

## 4. Therapeutic Applications of MSCs in Companion and Farm Animals

The therapeutic development of MSCs has progressed unevenly across veterinary species and disease categories [18,19]. Controlled clinical evidence is concentrated mainly in naturally occurring canine osteoarthritis, whereas equine tendon and joint studies are more heterogeneous and many neurological, cardiovascular, reproductive, wound healing, and production animal applications remain investigational or preclinical [19,94]. Evidence from experimentally induced lesions, tissue engineering models or in vitro systems should therefore not be treated as equivalent to clinical efficacy in animals with naturally occurring disease [18,19,20].

Interpretation is further complicated by differences among MSC products and treatment protocols [3,4,19,23,27]. Tissue source, donor selection, autologous or allogeneic use, passage, cryopreservation, dose, route, formulation, biomaterial combinations, and concurrent treatments can each influence outcome [3,4,27,34,35,36,50,51,52,53,54,55]. Clinical improvement must also be distinguished from structural regeneration because reduced pain, lameness, or inflammatory biomarkers does not necessarily demonstrate restoration of normal cartilage, tendon, neural tissue, or reproductive function [18,19,94]. Across indications, the strength of evidence should therefore be interpreted according to product definition, study design and controls, sample size, objective outcomes, duration of follow-up, and reported safety [18,19,23].

### 4.1. Musculoskeletal and Orthopaedic Disorders

Musculoskeletal disease is the most extensively investigated veterinary application of MSC therapy [18,19,94]. Horses and dogs develop naturally occurring tendon, ligament, and joint disorders that are clinically important and may provide comparative models for related human conditions [19,20].

#### 4.1.1. Tendon and Ligament Injuries

Equine superficial digital flexor tendon injury was among the earliest naturally occurring veterinary conditions treated with culture-expanded MSCs. Initial work demonstrated the feasibility of isolating autologous bone marrow-derived MSCs and implanting them directly into tendon lesions, but it was not designed to establish comparative efficacy [95]. A subsequent cohort study associated intralesional bone marrow-derived MSC administration with improved long-term outcome and a lower reinjury rate than historical expectations, although the non-randomized design limited causal inference [96].

A randomized, controlled, and triple-blinded pilot trial later evaluated allogeneic multipotent stromal cells in horses with naturally occurring tendon disease and supported the feasibility of prospective controlled investigation. The trial was exploratory and was not sufficiently powered to establish definitive efficacy, product superiority, or an optimal treatment protocol [97]. A 2026 systematic review and meta-analysis of 17 studies found that return to soundness or performance, lameness, ultrasonographic tissue characteristics, and microstructure generally favoured MSC treatment, whereas tissue gene expression, composition, and mechanical properties did not consistently favour MSCs over controls [94].

Return to athletic function and reinjury after return to work are clinically important endpoints, but they are influenced by lesion type, discipline, rehabilitation, and follow-up duration [94,96,97]. Ultrasonographic appearance, fibre alignment, vascularity, and histological organization provide useful intermediate evidence, but improvement in these measures does not demonstrate restoration of normal tendon biomechanics. The absence of a consistent advantage in mechanical properties in the recent meta-analysis is particularly important when interpreting claims of complete tenogenic regeneration [94].

Bone marrow-derived MSCs remain the most extensively studied product for equine tendon injury, but direct adequately powered comparisons have not established their superiority over adipose-, blood-, tendon-, or perinatal tissue-derived products [94,95,96,97]. Combination treatment with platelet products, scaffolds, or structured rehabilitation can further confound attribution when the independent effects of each component are not isolated [18,94]. Product-specific conclusions are therefore more defensible than treating all equine MSC preparations as one therapeutic class [4,19,23,27].

Canine tendon and ligament evidence is much less developed [19]. A pilot study combined autologous MSC-related interventions with tibial tuberosity advancement in dogs with cranial cruciate ligament injury, but its small groups and surgical co-intervention prevented determination of an independent cellular effect [98]. Experimental tendon models and scaffold-assisted strategies may provide proof of concept, but they should remain classified as preclinical evidence until supported by controlled trials in naturally occurring disease [18,19,20].

Intralesional MSC treatment can therefore be described as clinically promising for selected equine tendon and ligament injuries [94]. Evidence supports improvements in several clinical and imaging outcomes, but product superiority, optimal dosing, rehabilitation interactions and recovery of normal tissue mechanics remain unresolved [94,97].

#### 4.1.2. Osteoarthritis and Cartilage Injury

Osteoarthritis is the veterinary indication with the largest body of controlled companion animal MSC evidence, particularly in dogs [19]. Randomized and masked trials have reported product-specific improvements in pain, lameness, mobility, or function after intra-articular administration, although responses are not uniform across products or outcome measures [19,99,100,101].

A randomized, masked, and placebo-controlled trial reported clinical benefit after intra-articular administration of allogeneic adipose-derived MSCs in dogs with osteoarthritis [99]. In contrast, a small exploratory trial comparing repeated allogeneic MSC injections with high-molecular-weight hyaluronic acid did not demonstrate MSC superiority; improvements were inconsistent, the hyaluronic acid group performed better on several measures, and transient joint flares occurred in some MSC-treated dogs [100]. A separate randomized placebo-controlled trial of equine umbilical cord-derived MSCs in dogs with naturally occurring osteoarthritis reported improvements in selected pain and functional outcomes, but its xenogeneic product should not be generalized to autologous or allogeneic canine MSCs [101].

Outcome interpretation depends on the joint affected, disease severity, cell product, dose, assessment time, and measurement method [19,99,100,101]. Owner questionnaires, clinician-assessed lameness, activity monitoring, and force platform measurements capture different constructs and should not be treated as interchangeable [19,99,100]. Rescue analgesia, rehabilitation, hyaluronic acid, and platelet-derived products can also affect pain and function and should be controlled or reported transparently [18,19].

Most canine trials primarily demonstrate symptomatic or functional change rather than complete structural regeneration [19,99,100,101]. Reduced pain or improved weight bearing does not establish restoration of normal hyaline cartilage, and imaging, arthroscopy, biomarkers, and histology should be reported separately from clinical outcomes [18,19]. A cautious reading is that selected MSC products may improve symptoms and modulate the intra-articular environment in some dogs without proving disease cure or complete cartilage replacement [4,6,19].

Equine evidence includes naturally occurring stifle and meniscal disease as well as experimental synovitis and cartilage injury models [19,102]. A prospective clinical series reported favourable outcomes after intra-articular bone marrow-derived MSC administration in horses with stifle injury, but the uncontrolled design limits efficacy conclusions [102]. Experimental equine models provide mechanistic and safety information but do not establish effectiveness in chronic naturally occurring osteoarthritis [18,19].

Intra-articular MSC administration is often tolerated, but transient post-injection inflammation and product-specific immune responses have been reported. Autologous, allogeneic, and xenogeneic products should not be considered interchangeable because donor compatibility, repeated administration, and species origin may alter immunological risk [9,50,99,100,101]. Long-term safety assessment should include repeated-dose reactions and durability of benefit [50,99,100].

Current evidence supports product-specific improvement in pain or function in some dogs with osteoarthritis, but it does not support universal efficacy, disease cure, or complete cartilage regeneration [19,99,100,101]. Equine intra-articular therapy remains clinically promising but is supported by less high-level controlled evidence and greater product heterogeneity [19,102].

#### 4.1.3. Equine Laminitis

MSC-based treatment has been explored in horses with chronic laminitis, but evidence remains limited to small, uncontrolled clinical reports. Dryden et al. described the use of umbilical cord blood- and bone marrow-derived MSC preparations in affected horses, although the brief report provides insufficient methodological detail for firm interpretation. Angelone et al. treated nine horses with severe chronic laminitis using adipose-derived MSCs combined with platelet-rich plasma and reported improvements in clinical findings, hoof vascularization, and function. Because this study lacked a control group and used a combination treatment, the independent contribution of MSCs cannot be determined. Equine laminitis should therefore remain an investigational indication requiring controlled studies with standardized disease classification, objective hoof imaging, pain and locomotion outcomes, and long-term evaluation of recurrence and return to function [103,104].

#### 4.1.4. Bone Repair

MSCs have been combined with ceramics, hydroxyapatite, collagen, synthetic polymers, hydrogels, and other matrices to support bone regeneration in large animal models [20,105,106,107]. Such models are valuable because defect dimensions, loading conditions, and implant scale may be more clinically relevant than those in rodents, but they remain experimental models rather than proof of routine veterinary efficacy [20].

Allogeneic MSCs incorporated into a scaffold promoted bone formation in a critical-sized canine segmental defect [105]. Autologous bone marrow stromal cells loaded onto porous hydroxyapatite accelerated repair in critical-sized ovine long-bone defects [106]. More recent work evaluated poly-L-lactic acid/graphene oxide scaffolds filled with canine placental hydrogel and MSCs in goat mandibular defects, illustrating the increasing complexity of combination constructs [107].

These studies provide translational proof of concept but do not establish routine effectiveness in naturally occurring fractures, delayed unions or non-unions [20,105,106,107]. Increased mineralization or new bone formation is not equivalent to restoration of normal mechanical competence, so evaluation should include vascularization, integration, cortical remodelling, biomechanical strength, and long-term load bearing [18,20]. The scaffold itself may recruit endogenous cells or modify inflammation and matrix formation, requiring scaffold-only, cell-only, and combined-treatment controls [18,23].

Bone repair remains a promising preclinical tissue engineering application rather than an established veterinary MSC therapy [20,105,106,107]. Clinical translation will require standardized constructs, indication-specific controls, mechanical endpoints, and reproducible manufacturing [20,22,23].

### 4.2. Wound Healing, Skin, and Ocular Repair

#### 4.2.1. Cutaneous Wounds

MSCs and MSC-derived products have been investigated in wounds because they can modify inflammation, angiogenesis, fibroblast activity, epithelial repair, and extracellular matrix remodelling [6,12,18,85,86]. Most veterinary evidence nevertheless derives from experimentally created wounds rather than naturally occurring chronic lesions [19].

In controlled equine distal-limb wound models, allogeneic MSC administration altered local gene expression and improved selected aspects of tissue repair [108]. A large animal study that combined platelet concentrate with allogeneic MSCs reported changes in healing quality, but the biologically active platelet product prevented isolation of an independent MSC effect [109]. Combination studies therefore require platelet-only, cell-only, and combined-treatment controls when attribution is a study objective [18,23].

A pilot study tested MSC-conditioned medium in methicillin-resistant Staphylococcus aureus-inoculated equine thoracic wounds. The conditioned medium did not reduce bacterial colony counts, wound size, gross wound scores, or microscopic wound scores relative to vehicle under the tested conditions, whereas mupirocin-treated wounds were smaller at the final assessment. These negative findings show that in vitro antimicrobial or trophic activity cannot be assumed to translate into efficacy in an infected in vivo wound model [110].

In Bama miniature pigs with experimentally induced deep partial-thickness burns, human umbilical cord MSC treatment accelerated healing in a dose-related experimental setting [111]. This xenogeneic burn model is useful for studying dose and tissue response but differs substantially from naturally occurring chronic wounds complicated by vascular disease, infection, age or repeated trauma [20,111].

MSC-based interventions may improve selected healing outcomes in controlled experimental wounds, but clinical evidence in naturally occurring canine, equine, and livestock wounds remains preliminary [19,108,109,110,111]. Future trials should assess epithelial integrity, infection control, scar quality, recurrence, and long-term function rather than wound area alone [18,23].

#### 4.2.2. Ocular Disease

Ocular applications permit local delivery and direct clinical monitoring, but product retention, corneal transparency, neovascularization, and concurrent topical therapy can influence outcome [19]. Proposed benefits include immunomodulation, support of epithelial repair, and modulation of fibrosis, but these mechanisms require product- and indication-specific validation [4,6,19].

In dogs with naturally occurring keratoconjunctivitis sicca, topical allogeneic adipose-derived MSC administration improved selected tear-film and ocular-surface measures. This study provides promising clinical evidence, but larger masked and independently replicated trials are needed to define durability, responder characteristics, and effects on vision [112]. The treatment is best described as investigational rather than established standard care [19,112].

An equine case report described corneal healing after application of an MSC secretome to a non-healing ulcer. A single case can demonstrate feasibility but cannot establish efficacy because spontaneous recovery, lesion heterogeneity, and concurrent therapy cannot be controlled. Equine corneal secretome therapy therefore remains case-based and requires controlled evaluation [113].

### 4.3. Neurological and Neuromuscular Disorders

Neurological injury may involve neuronal or axonal loss, demyelination, inflammation, vascular compromise, and glial scarring, making a single regenerative mechanism unlikely to address all disease components [6,19]. The most plausible MSC contributions are neurotrophic, immunomodulatory, angiogenic, and cytoprotective rather than direct replacement by mature, functionally integrated neurons [4,6].

An experimental equine peripheral nerve injury model evaluated local MSC administration and long-term recovery, providing preliminary evidence of feasibility and selected regenerative effects [114]. In companion animals with chronic spinal cord injury, combined intrathecal and intravenous administration of bone marrow stromal cells with platelet-rich plasma was associated with clinical improvement in a small heterogeneous cohort. Interpretation is limited because platelet products, baseline injury severity, spontaneous recovery, surgery, and rehabilitation can each influence neurological outcome [115].

A canine preclinical study combined bone marrow aspirate concentrate with an ultra-purified alginate gel and reported improved intervertebral disc repair measures. Because the intervention combined cellular material with a bioactive delivery matrix in an experimental model, it does not establish clinical efficacy for naturally occurring intervertebral disc disease [116]. Appropriate translation would require matrix-only, cell-only, and combined controls as well as neurological and functional endpoints [18,23,116].

Expression of neural markers or neuron-like morphology does not demonstrate electrophysiological integration, axonal connectivity or restoration of neurological function [6,30,117]. Current veterinary reviews consequently classify cellular therapies for neurological disease in dogs and horses as investigational [117]. Future studies require randomization, injury stratification, standard-care or sham controls, validated neurological scores, imaging, electrophysiology, and long-term follow-up [18,23,117].

### 4.4. Cardiovascular and Ischaemic Injury

Pigs have been widely used as large animal models of MSC treatment for myocardial infarction and chronic ischaemic cardiomyopathy because their cardiac anatomy and suitability for catheter-based or surgical intervention permit clinically relevant delivery and imaging studies. These studies are primarily translational models for human cardiovascular medicine rather than established treatments for veterinary cardiac patients [20].

Early porcine studies reported engraftment and functional effects after MSC implantation following myocardial infarction [118]. Subsequent work associated intramyocardial administration of allogeneic MSCs with favourable structural or functional cardiac outcomes [119]. MSCs were also evaluated in chronic ischaemic cardiomyopathy, with improvements reported in selected cardiac-function measures [120].

Early interpretations sometimes emphasized cardiomyogenic differentiation, but limited persistence and broader mechanistic evidence support major roles for trophic, angiogenic and immunomodulatory activity [6,7,12,118,119,120]. Expression of cardiac-associated markers is not equivalent to formation of mature, electrically integrated cardiomyocytes [6,30]. Arrhythmia, microvascular obstruction, delivery injury, cell retention and durability remain important translational concerns [20,118,119,120].

The porcine cardiovascular literature is best presented as large animal preclinical evidence, not as proof of a routine veterinary cardiac indication [20,118,119,120]. Clinical veterinary use would require disease-specific safety, efficacy and delivery studies in naturally affected animals [18,23].

### 4.5. Immune-Mediated and Inflammatory Diseases

Naturally occurring inflammatory diseases in dogs and cats permit investigation of MSC therapy in genetically diverse animals receiving clinically relevant care [19]. Evidence is promising for selected refractory diseases but remains dependent on the indication, product, concurrent medication and host inflammatory state [7,19].

Allogeneic adipose-derived MSCs have been evaluated in dogs with chronic inflammatory enteropathy, with improvements reported in selected clinical and laboratory outcomes [121]. Later work compared outcomes in dogs treated with MSCs with or without corticosteroids, highlighting the difficulty of separating cellular effects from concurrent immunosuppression [122]. Sample sizes were modest, and differences in diet, disease phenotype, medication and follow-up limit generalization [121,122].

A double-blinded placebo-controlled study reported improvement in selected dogs with atopic dermatitis after adipose-derived MSC administration. The magnitude and duration of benefit require independent replication, and MSC treatment should not be presented as a replacement for established allergen management or immunomodulatory therapy [123].

Feline chronic gingivostomatitis illustrates the importance of treatment sequence and disease stage [124,125]. MSC administration before full-mouth tooth extraction did not produce substantial clinical efficacy [124]. In contrast, longer-term open-label experience in cats with refractory disease after conventional treatment identified durable responders, although responses remained heterogeneous and the absence of a concurrent control limited inference [125].

Selected canine and feline inflammatory diseases can therefore be described as clinically promising but not routinely established indications [19,121,122,123,124,125]. Future trials should standardize disease classification, concurrent treatment, validated clinical indices, relapse monitoring and biomarkers of recipient or product response [4,19,23,27].

### 4.6. Reproductive Applications

#### Uterine Disease and Fertility

In mares, persistent post-breeding-induced endometritis and chronic degenerative endometritis, or endometrosis, are biologically and clinically distinct disorders. Persistent post-breeding-induced endometritis results from failure to resolve mating-associated uterine inflammation, whereas endometrosis is characterized by progressive fibrosis, glandular degeneration and structural deterioration of the endometrium [59,126,127].

Amniotic MSC-derived extracellular vesicles have been investigated in mares as a semen-associated intervention to reduce post-breeding inflammation. Although anti-inflammatory activity was reported, this study did not establish improvements in conception, pregnancy maintenance or live-foal outcome and should not be interpreted as evidence that EVs reverse chronic endometrial fibrosis [126].

A 2026 uncontrolled case series evaluated intrauterine amniotic cell-derived EVs in 12 mares with histologically confirmed endometrosis. Eleven mares became pregnant after treatment, but the small cohort, absence of a control group and reliance on previous reproductive failure limit causal interpretation. Moreover, the Kenney–Doig endometrial classification did not improve, indicating that histological reversal of the degenerative condition was not demonstrated [127].

In sheep, a bioengineered tissue patch promoted repair of an experimentally created uterine defect. This finding provides proof of concept for reproductive tissue engineering but cannot be extrapolated directly to naturally occurring equine endometritis, endometrosis or infertility. Clinically relevant outcomes should include pregnancy, live-offspring production, placental function and reproductive safety rather than short-term histological or inflammatory changes alone [128].

MSC- and EV-based reproductive interventions should therefore remain classified as investigational. Current evidence supports selected anti-inflammatory and tissue-repair effects, but consistent improvement in fertility, durable reversal of fibrosis and offspring safety have not yet been established [59,126,127,128].

### 4.7. Mammary Gland and Mastitis

Mastitis is an important inflammatory and infectious disease of dairy cattle, and MSCs have been proposed as adjunctive treatments because of their immunomodulatory and trophic properties [21,129,130]. Intramammary MSC administration has been evaluated in cows with experimentally induced Staphylococcus aureus mastitis, with feasibility and selected clinical or inflammatory changes reported. Experimental infection does not fully reproduce the pathogen diversity, chronicity, treatment history and management conditions of naturally occurring mastitis [129].

A 2024 study evaluated allogeneic bone marrow- and adipose-derived cells in dairy cows classified largely through evidence of subclinical udder inflammation. This study does not constitute definitive treatment evidence for naturally occurring acute clinical mastitis, and its relatively short follow-up limits conclusions about recurrence, milk production and long-term udder recovery [130]. Cell source and product differences also prevent pooling of its results with the experimental Staphylococcus aureus study [129,130].

Reduction in inflammatory indices does not establish bacteriological cure, restoration of milk yield or prevention of recurrence [129,130]. Future studies should assess pathogen clearance, somatic cell counts, milk production and composition, udder structure, recurrence, rescue antimicrobial use and adverse events [21,129,130]. Translation to food-producing animals additionally requires jurisdiction-specific assessment of manufacturing controls, biosafety, milk disposition, withdrawal requirements and economic feasibility [23,24,25,129,130].

Bovine MSC therapy for mastitis should therefore remain classified as investigational. Comparative effectiveness against antimicrobial and supportive care, food chain safety and cost-effectiveness remain unresolved [21,129,130].

### 4.8. Tissue Engineering and Biomaterial-Assisted Delivery

Poor retention and survival of administered MSCs remain major limitations of local cell therapy. Cells may be lost through leakage, mechanical displacement or inadequate attachment, while inflammation, hypoxia, nutrient deprivation and extracellular matrix disruption can further reduce viability after implantation. Hydrogels, scaffolds, encapsulation systems and decellularized matrices have therefore been developed to retain MSCs at the target site, protect them during delivery and provide a supportive microenvironment [20,23,131].

These materials do more than serve as physical carriers. Their stiffness, ligand density, porosity, fibre orientation, viscoelasticity and degradation rate can influence MSC adhesion, survival, secretion and lineage-associated programmes through mechanotransduction. Biomaterial design may therefore enhance retention and functional activity, but an unsuitable construct may restrict nutrient exchange, provoke inflammation or degrade too rapidly or too slowly [23,63,64,131].

Micromoulded alginate encapsulation has been investigated for intra-articular MSC delivery, while bone, intervertebral disc and uterine models demonstrate that scaffold composition and architecture can materially alter the biological response [105,106,107,116,128,131]. Because both the cells and the material can contribute independently to repair, MSC–biomaterial constructs should be evaluated as combination products. Studies should include material-only, cell-only, and combined-treatment groups and assess cell retention, viability, integration, degradation, mechanical performance, and long-term safety [18,20,22,23,131].

Biomaterial-assisted delivery remains a promising preclinical strategy for bone, cartilage, tendon, skin, nerve, and uterine repair. Clinical translation will require reproducible manufacturing and evidence that the combined construct provides a measurable advantage over either the MSC product or the biomaterial alone [20,22,23,105,106,107,116,128,131].

### 4.9. Cell-Free Therapeutic Applications

MSC-derived conditioned medium, concentrated or freeze-dried secretome and EV preparations require separate evaluation because their composition, manufacturing, dosing, and biological behaviour differ from those of viable cells. Cell-free products should not be treated as a uniform class or assumed to be equivalent to their parental MSCs [15,16,18,19,56].

A canine MSC lyosecretome was evaluated for pharmaceutical production feasibility and intra-articular administration in a very small number of osteoarthritic dogs. This study primarily supported manufacturing feasibility and preliminary tolerability and did not provide robust evidence of efficacy [132]. A later randomized, double-blinded controlled study in 26 dogs reported improvement in selected outcomes after a formulation-specific lyosecretome treatment, while five horses contributed preliminary safety observations [133]. These findings support continued evaluation of that defined product but should not be generalized to other conditioned media, secretomes, or EV preparations [132,133].

Most veterinary EV evidence remains preclinical or in vitro [15,16,19]. Equine bone marrow MSC-derived EVs were taken up by autologous chondrocytes and produced anti-inflammatory effects in vitro, but this does not establish efficacy after intra-articular administration in diseased horses [134]. Similarly, reproductive and ocular findings are product- and indication-specific [113,126,127].

Cell-free preparations require explicit reporting of producer cells, culture medium, collection interval, concentration, separation, storage, dose, contaminants, and indication-relevant potency. EV terminology and characterization should follow MISEV2023 guidance, including evaluation of serum-derived particles when vesicle-containing supplements are used [16]. Manufacturing differences can change cargo and activity, so equal particle number or total protein does not establish functional equivalence [15,16,18,19,56].

Cell-free products may simplify some aspects of storage and avoid administration of large viable cells, but their biodistribution, immunogenicity, contaminants, off-target effects, and long-term safety still require direct testing [15,16,18,19,56]. Unlike viable MSCs, a manufactured cell-free preparation cannot dynamically alter its secretory response after administration [15,16]. Cell-free MSC therapeutics should therefore be regarded as an emerging, heterogeneous and formulation-specific product class [15,16,18,19,56,132,133,134].

### 4.10. Critical Interpretation of the Therapeutic Evidence

Veterinary MSC evidence is neither uniformly positive nor uniformly inconclusive; it is indication-, product-, and study-design specific [18,19,23]. The strongest controlled companion animal evidence is concentrated in canine osteoarthritis, where selected products improved pain or function, but an active comparator trial did not demonstrate MSC superiority [19,99,100,101]. Equine tendon and ligament evidence increasingly supports clinical, ultrasonographic, and microstructural benefit, although tissue composition and mechanical properties remain inconsistent [94]. Equine joint evidence is promising but relies more heavily on uncontrolled series and experimental models [19,102]. Equine laminitis remains supported only by small uncontrolled reports and combination treatment [103,104].

Selected inflammatory diseases in dogs and cats also show potential, but chronic enteropathy, atopic dermatitis, and feline gingivostomatitis require independent replication, better control of concurrent treatment, and identification of responder characteristics [121,122,123,124,125]. Bone repair, wounds, neurological and cardiovascular applications, uterine therapy, and most biomaterial constructs remain predominantly preclinical or investigational [20,105,106,107,108,109,110,111,112,113,114,115,116,117,118,119,120,121,122,123,124,125,126,127,128,129,130,131]. Negative studies, including the equine infected wound secretome experiment, are essential because they demonstrate that mechanistic plausibility and in vitro activity do not guarantee in vivo efficacy [110].

Interpretation is repeatedly limited by small cohorts, inconsistent randomization or masking, incomplete product characterization, heterogeneous dosing and storage, confounding co-interventions, and insufficient long-term safety assessment [4,19,20,23,27,94,99,100,101,102]. Symptomatic improvement should be separated from structural or mechanical regeneration [19,94,99,100,101,102]. Autologous, allogeneic, and xenogeneic products should not be pooled as biologically equivalent [9,50,99,100,101]. The more useful question is which defined MSC or MSC-derived product benefits a particular disease, species, and stage under a specified manufacturing, dosing and delivery process [4,19,23,27]. The evidence and translational maturity of the principal therapeutic applications are summarized in Table 2.

## 5. Comparative Translational Roles of Veterinary Species

Veterinary species contribute to MSC research through complementary clinical, experimental, and agricultural roles. Companion animals provide naturally occurring disease, large experimental animals enable human-scale procedures and biomaterial testing, and production livestock contribute reproductive, mammary, and herd-level biological systems. Translational value should therefore be judged by fitness for purpose rather than by body size, publication count, or an assumed hierarchy of similarity to humans [135,136]. Their complementary translational roles are summarized in Table 3.

### 5.1. Companion Animals as Naturally Occurring Clinical Systems

Dogs, horses, and cats develop spontaneous disease in genetically and environmentally heterogeneous populations receiving realistic diagnostic, medical, surgical, and rehabilitative care. This complexity incorporates ageing, chronicity, comorbidity, and previous treatment, but also increases variability. Naturally occurring disease should therefore be considered a comparative clinical system only when the relevant pathogenesis, anatomy, immune response, and outcome measures are sufficiently aligned with the question under study [19,135].

Dogs provide the broadest controlled companion animal MSC evidence, particularly in osteoarthritis [19,99,100,101]. Canine disease permits assessment of pain, mobility, weight bearing, and quality of life, but results remain product-specific and are not uniformly positive [100]. Chronic inflammatory enteropathy, atopic dermatitis, and neurological or intervertebral disc studies extend the comparative value of dogs, although evidence outside osteoarthritis remains smaller and more exploratory [116,117,121,122,123].

Horses are especially informative for high-load tendon, ligament, and joint disorders because rehabilitation, return to athletic function, and reinjury can be followed under substantial mechanical demand [94,95,96,97,102]. Pooled evidence favours several clinical and imaging outcomes in equine tendinopathy and desmopathy but does not consistently demonstrate restoration of tissue composition or mechanics. Athletic discipline, lesion location, and rehabilitation materially influence outcomes, so horses are most useful for defined biomechanical and functional questions rather than as universally superior musculoskeletal models [94].

Feline evidence is narrower. Chronic gingivostomatitis has produced contrasting outcomes according to disease stage and treatment sequence, with limited benefit before full-mouth extraction but durable responses in a subset of refractory cats treated after conventional management. Cats therefore provide a focused system for selected chronic inflammatory diseases rather than a mature general platform for MSC efficacy [124,125].

### 5.2. Procedure-, Device-, and Biomaterial-Oriented Large Animal Models

Pigs support catheter-based delivery, surgery, advanced imaging, physiological monitoring, and clinically relevant device testing because of their organ size and cardiovascular anatomy [137]. Porcine myocardial infarction and chronic ischaemia models have enabled evaluation of intramyocardial delivery, cell retention, and ventricular function. These models are strongest for procedural feasibility, dose, biodistribution, and biological plausibility, but acute injury in young healthy animals does not reproduce ageing, chronic comorbidity, or the full immune and coagulation context of human cardiac disease [118,119,120].

Comparative studies also show that human, ovine and porcine bone marrow MSCs differ in proliferation, phenotype, and differentiation, reinforcing the need for species-specific product characterization [48]. Sheep and goats are valuable for load-bearing orthopaedic and tissue engineering studies because their skeletal dimensions permit clinically relevant defects, fixation systems and constructs. Their main strengths are feasibility, scaffold integration, degradation, and mechanical performance; standardized defects in young healthy animals are less predictive of chronic degenerative disease [106,107,138,139].

Beyond therapeutic efficacy studies, large animal models can support biodistribution, device, biomaterial, and toxicological evaluation under anatomical and physiological conditions that are difficult to reproduce in rodents or conventional cell culture. Their value nevertheless remains application-specific, and findings should be interpreted according to species differences, model design, and the extent to which the experimental system reproduces the intended clinical setting [22,137].

### 5.3. Production Livestock as Agricultural and Functional Systems

Cattle and buffalo function both as research models and as target species for reproductive, mammary and production-relevant applications. In these settings, economic feasibility, scalability, food chain safety, and herd-level outcomes are central and differ substantially from individualized companion animal therapy [23,24,25,136].

Bovine studies have progressed from characterization towards mastitis and reproductive biology. Umbilical cord blood MSCs were evaluated in cows with subclinical mastitis, but product-specific reductions in inflammatory measures should not be generalized to all pathogens or interpreted automatically as bacteriological cure or sustained milk recovery [140]. Experimental work indicates that MSCs can modify uterine and mammary infection or inflammation, although much of this evidence derives from cell culture and murine systems rather than therapeutic trials in cattle [141]. Trophectoderm secretomics studies further support embryo–maternal communication involving endometrial and circulating stromal cells but do not demonstrate that exogenous MSC administration improves fertility [142].

Buffalo research remains dominated by source characterization, culture optimization, hypoxia, and cryopreservation [25,26,52]. A small single-centre study of allogeneic adipose-derived MSCs in chronic endometritis reported improved inflammatory and reproductive outcomes, providing preliminary direct evidence that requires independent controlled replication [143].

### 5.4. Fitness-for-Purpose Model Selection and Bidirectional Translation

No veterinary species is optimal for every regenerative question. Model selection should begin with the intended endpoint and then consider disease similarity, anatomy, age, immune and coagulation context, product compatibility, administration route, and measurable outcomes. Naturally occurring disease offers clinical realism but introduces heterogeneity, whereas induced models improve experimental control but may omit chronicity and comorbidity [20,48,135,136,137,138,139]. Transparent planning, appropriate controls, and complete reporting are therefore as important as species selection [18,19,23].

Translation is bidirectional rather than a one-way progression from animals to humans. Human cell therapy research contributes manufacturing, potency, and regulatory principles, while veterinary patients and livestock provide spontaneous disease, repeated allogeneic exposure, human-scale procedures, and agricultural systems unavailable in conventional rodent models. A One Health interpretation is most defensible when reciprocal clinical, welfare or agricultural benefits are explicit. Comparative programmes should therefore preserve direct justification for the animal species involved and avoid treating veterinary patients or livestock merely as substitutes for human experimentation [19,20,135,136].

## 6. Future Perspectives and Clinical Translation

**Figure 2:**
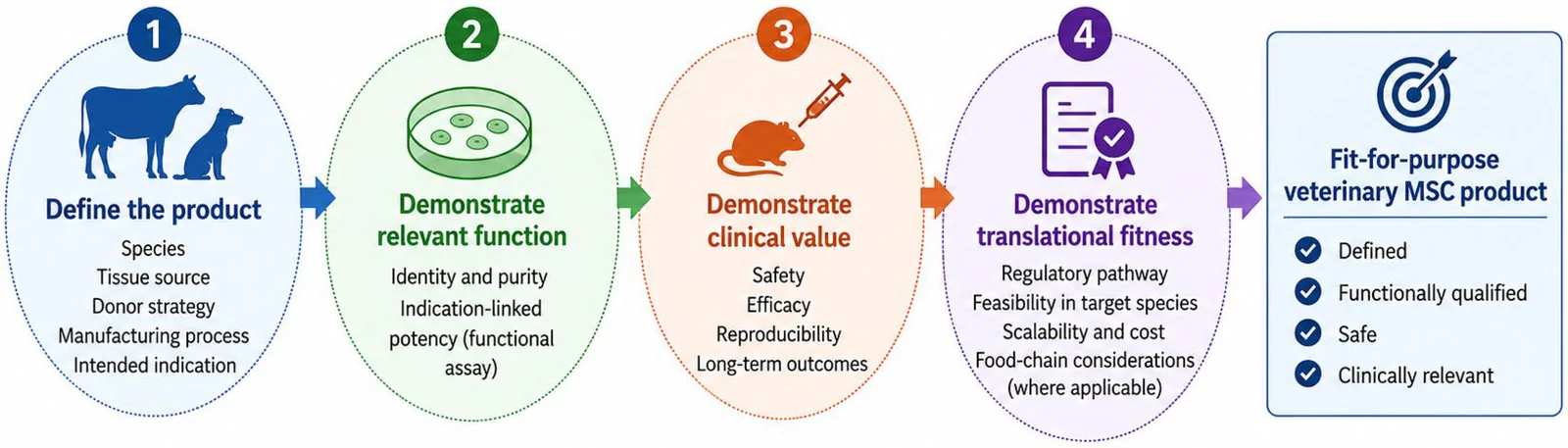
Translational framework for veterinary mesenchymal MSC and MSC-derived products. Translation requires alignment of product identity with its intended clinical use. A defined product, specified by species, tissue source, donor strategy, and manufacturing process, should demonstrate indication-relevant functional potency before clinical value is assessed through safety, efficacy, reproducibility, and long-term outcomes. Further translation depends on regulatory requirements and practical feasibility, including scalability, cost, and, where relevant, food chain considerations. The framework emphasizes that species, product characteristics, and intended indication should be evaluated together rather than treating MSCs as a uniform therapeutic product. This figure was generated using ChatGPT (OpenAI, GPT-5.6 Sol) based on a conceptual framework conceived by the authors. The scientific content, pathway relationships, labels, and final presentation were reviewed and edited by the authors.

The next phase of veterinary MSC research should move beyond proof-of-concept isolation and broadly characterized cell preparations towards reproducible, indication-specific products. Translation requires coordinated development of potency testing, manufacturing control, clinical evidence, safety surveillance, and regulation rather than isolated optimization of individual components [19,23,27,144,145,146,147,148,149]. The principal stages of product development and clinical implementation are summarized in Figure 2.

### 6.1. Product Definition, Potency, and Manufacturing

Conventional morphology, plastic adherence, immunophenotyping, and trilineage differentiation support MSC identity but do not establish efficacy for a particular indication. A translational product should therefore be defined by species, donor strategy, tissue and anatomical source, expansion history, formulation, storage, viable dose, route of administration, and intended biological activity [1,2,144].

Potency assays should measure functions linked to the proposed mechanism and clinical use. T-cell suppression assays may be appropriate for immunomodulatory products but cannot predict cartilage protection, angiogenesis, antimicrobial support, or tendon repair. A qualified matrix combining complementary cellular, molecular, secretory, or metabolic measurements is therefore more defensible than reliance on a single marker, provided that the assays remain reproducible and practical for routine lot release [145,146,147].

Manufacturing is itself a determinant of biological activity. Donor health, tissue source, cumulative expansion, culture medium, oxygen tension, cryopreservation, formulation, and delivery method can alter proliferation, senescence, secretion, immunomodulation, and haemocompatibility. Process changes should therefore be supported by comparability testing, while release testing should include identity, purity, viable dose, sterility, mycoplasma, endotoxin, adventitious agent control, and indication-linked potency. Intravascular products additionally require assessment of cell size, tissue factor expression, dose, and route because thrombo-inflammatory risk is product-dependent [27,34,35,36,49,50,51,52,53,54,55,144,148,149,150,151,152].

Primed MSCs, conditioned medium, lyosecretome, extracellular vesicle products and biomaterial-assisted constructs should be treated as distinct product classes rather than automatic improvements over conventional MSCs. Their composition and activity depend on manufacturing conditions, and unresolved challenges include dose normalization, stability, contaminants, biodistribution, haemocompatibility, and batch consistency. These products should therefore be compared directly with appropriately qualified conventional controls rather than presumed equivalent or superior [15,16,131,132,133,134,153,154,155].

### 6.2. Clinical Evidence, Safety, and Feasibility

Veterinary trials should progress towards multicentre designs using harmonized products, prespecified sample-size calculations, appropriate comparators, allocation concealment, and masking where feasible. Product identity, cumulative expansion, formulation, storage, thawing, dose, and concurrent treatment should be reported sufficiently for replication. Subjective clinical assessments should be paired with objective measures such as gait analysis, imaging, histology, biomarkers, or predefined rescue medication criteria [19,23,156,157].

Short-term tolerability has generally been acceptable for several defined canine and equine products, but MSCs should not be considered universally immune privileged. Repeated or MHC-mismatched allogeneic administration can induce humoral and cellular responses, and route-specific safety findings cannot be generalized across products or indications. Safety assessment should therefore include contamination, traceability, acute inflammatory reactions, haemocompatibility, immune sensitization, genomic instability, senescence, and delayed adverse events. Neoplasia and ectopic tissue formation remain appropriate surveillance outcomes, although neither has emerged as a frequent complication in the limited veterinary cohorts available to date [9,50,152,156,158,159,160,161,162,163].

Economic feasibility must also be evaluated according to the intended setting. Autologous manufacture is constrained by delay, donor health and batch failure, whereas allogeneic banking improves availability but adds donor qualification, cell bank testing, cold chain, and immunogenicity costs. In food-producing animals, scalability, milk or meat safety, withdrawal considerations, and herd economics create additional barriers to routine application [23,24,25,148,149,163].

### 6.3. Regulatory Translation and Outlook

Veterinary regulation remains jurisdiction-specific. In the United States, relevant animal cell and tissue products may fall within the new animal drug framework described in FDA Guidance for Industry documents addressing product classification, manufacturing practice, and donor eligibility. In the European Union, veterinary cell-based products are governed by the broader veterinary medicinal product framework together with guidance on potency, target animal safety, sterility, extraneous agents, and tumorigenicity. Regulatory review or enforcement discretion should not be confused with formal marketing authorization [164,165,166,167,168,169,170,171,172,173,174].

International convergence is most realistic for terminology, traceability, donor qualification, critical quality attributes, comparability, and adverse-event reporting rather than identical approval pathways. Progress will ultimately depend less on generating additional broadly characterized MSC preparations than on developing disease-, species-, and product-specific therapies whose quality, safety, feasibility, and clinical value can be independently verified. Comparative translation should remain bidirectional, with human cell therapy standards strengthening veterinary development while naturally occurring disease, repeated allogeneic treatment, human-scale procedures, and livestock systems provide evidence unavailable from conventional laboratory models [19,20,23,27,135,136,144,145,146,147,148,149,163].

Emerging technologies may further improve MSC characterization and product development. Transcriptomic, proteomic, metabolomic, and single-cell approaches can reveal cellular heterogeneity and molecular states not captured by conventional markers [35]. Molecular engineering and targeted preconditioning may be used to modify selected MSC functions, although their effects on product stability, safety, and manufacturing consistency require careful evaluation [163,175]. Artificial intelligence and computational approaches may also assist in analysing complex molecular and imaging data and identifying features associated with MSC potency, although their usefulness will depend on standardized and well-annotated datasets [176]. Future studies should ultimately link these emerging technologies with indication-specific potency and therapeutic outcomes rather than using them only for descriptive characterization.

## 7. Conclusions

Veterinary MSC research has moved beyond demonstrating that MSC-like cells can be isolated and expanded from different tissues. The more relevant question is whether a defined MSC product can perform a defined biological function in a defined disease environment. Current evidence indicates that MSC activity is mediated mainly through context-dependent interactions with the host, including inflammatory licensing, immune cell regulation, soluble mediators, extracellular vesicles, metabolic adaptation, mechanotransduction, and responses to short-lived or apoptotic cells. However, the relative contribution of these mechanisms remains poorly resolved for most veterinary products and species.

Clinical translation is also less advanced than the volume of experimental literature may suggest. The strongest controlled evidence currently exists for selected MSC products in canine osteoarthritis, while equine tendon and joint applications remain promising but heterogeneous. Most neurological, cardiovascular, reproductive, wound healing, biomaterial-assisted, and production animal applications are still supported mainly by experimental models, small studies, or uncontrolled observations. Interpretation is further limited by heterogeneous products, variable dose and administration protocols, manufacturing and cryopreservation differences, concurrent treatments, small sample sizes, and inadequate long-term follow-up. Improvement in pain, inflammation, or short-term function should therefore not be equated automatically with structural regeneration or long-term disease modification.

A major unresolved problem is the lack of reliable predictors of therapeutic response. Conventional surface markers and trilineage differentiation support MSC identity but do not predict whether a product will suppress inflammation, promote matrix repair or provide another clinically relevant function. Future studies should determine which combinations of licensing response, secretome or EV composition, metabolic fitness, survival, biodistribution, and host cell responses are associated with therapeutic outcome, and whether these relationships are conserved across veterinary species.

## 8. Core Tips

Veterinary MSCs are heterogeneous products whose phenotype and functional potency vary with species, tissue source, donor, and manufacturing conditions.

MSC therapy is mediated primarily through host-directed immunomodulatory and paracrine mechanisms rather than long-term engraftment or tissue replacement.

Successful veterinary translation depends on standardized manufacturing, mechanism-based potency testing, and robust clinical evidence across species and indications.

## Figures and Tables

**Table 1:** Representative immunophenotypic characteristics of MSCs reported in veterinary species.

Species	Cell/Tissue Source	Reported Positive Markers	Reported Negative/Low or Variable Markers	References
Cattle	Bone marrow	CD73, CD90, CD105	CD34, CD45 low/negative	[38]
Buffalo	Bone marrow	CD73, CD90, CD105, STRO-1	—	[37]
Buffalo	Amniotic fluid/amniotic membrane	Stemness/mesenchymal-associated markers reported; marker profile differs with source	Variable depending on preparation	[42,43]
Dog	Adipose tissue/umbilical cord matrix	CD44, CD90	CD14 negative; CD34 weak/variable	[39]
Horse	Bone marrow/adipose tissue/umbilical cord	CD44, CD90, CD105 commonly reported	CD34 low/negative; MHC-II and some markers tissue-/passage-dependent	[31,32,33]
Pig	Bone marrow	CD29, CD44, CD90 commonly reported	Marker profile differs from human/ovine MSCs; antibody panel dependent	[48]

**Table 2:** Current evidence and translational maturity of major MSC-based applications in companion and farm animals.

Therapeutic Application	Species	Current Evidence Base	Proposed Principal Mechanisms	Translational Maturity *	Major Limitations	References
Osteoarthritis	Dog	Multiple randomized or masked controlled clinical trials	Synovial inflammatory modulation, immunoregulation, and trophic support	Moderate–High	Product heterogeneity; variable outcome measures; incomplete structural assessment; limited long-term follow-up	[20,99,100,101]
Tendon and ligament injury	Horse	Cohort studies, an exploratory randomized trial, systematic review, and meta-analysis	Inflammatory modulation, angiogenesis-associated repair, and extracellular matrix remodelling	Moderate	Heterogeneous products; variable rehabilitation; limited mechanical endpoints; inconsistent structural recovery	[94,95,96,97]
Joint disease	Horse	Experimental studies and uncontrolled or small clinical studies	Anti-inflammatory and trophic effects within the joint	Low–Moderate	Small cohorts; product heterogeneity; limited controlled evidence of structural regeneration	[20,102]
Equine laminitis	Horse	Small uncontrolled clinical reports and proof-of-concept combination treatment	Proposed inflammatory modulation and trophic or vascular support	Low	No controlled trials; limited methodological detail; confounding by platelet-rich plasma	[103,104]
Bone regeneration	Dog, sheep, and goat	Experimental critical-sized-defect and scaffold-based models	Osteogenic support, paracrine repair, and scaffold integration	Preclinical	Experimentally induced defects; complex combination products; limited evidence in naturally occurring disease	[105,106,107]
Cutaneous wound healing	Horse and pig	Controlled experimental wound models and pilot studies	Angiogenesis, inflammatory cell modulation, epithelial repair, and matrix remodelling	Preclinical–Low	Predominantly induced wounds; inconsistent efficacy; limited naturally occurring clinical evidence	[108,109,110,111]
Ocular disease	Dog and horse	Small clinical study and case-based evidence	Immunomodulation, epithelial support, and regulation of fibrosis	Low	Limited sample sizes; concurrent topical therapy; lack of independent replication	[112,113]
Neurological and neuromuscular disorders	Dog and horse	Experimental studies and small heterogeneous clinical cohorts	Neurotrophic support, cytoprotection, angiogenesis, and immunomodulation	Low	Heterogeneous disorders; combination treatments; limited objective functional evidence	[114,115,116,117]
Cardiovascular and ischaemic injury	Pig	Large animal preclinical myocardial infarction and chronic ischaemia models	Paracrine signalling, angiogenesis, cytoprotection, and inflammatory modulation	Preclinical	Experimentally induced injury; limited persistence; incomplete representation of chronic spontaneous disease	[22,118,119,120]
Chronic inflammatory enteropathy	Dog	Small clinical and comparative studies	T-cell regulation, inflammatory licensing, and innate immune modulation	Low–Moderate	Small cohorts; variable disease phenotypes; confounding by diet or concurrent corticosteroids	[121,122]
Atopic dermatitis	Dog	Early placebo-controlled clinical investigation	Immunomodulation and regulation of inflammatory responses	Low–Moderate	Limited replication; uncertain durability; incomplete identification of responders	[123]
Chronic gingivostomatitis	Cat	Pilot and open-label clinical studies	Immune regulation and pro-resolving activity	Low	Heterogeneous response; treatment-stage effects; absence of concurrent controls in longer-term studies	[124,125]
Uterine disease and fertility	Mare; experimental sheep model	Amniotic MSC-derived EV studies, uncontrolled equine case series, and experimental tissue engineering model	Anti-inflammatory EV signalling, tissue repair, and proposed modulation of fibrosis	Investigational	Small uncontrolled cohorts; distinct disease entities; limited fertility endpoints; no demonstrated reversal of endometrosis	[59,126,127,128]
Mastitis	Cattle	Experimental infection study and early field investigation	Inflammatory modulation and trophic support	Investigational	Limited controlled evidence; uncertain bacteriological cure; short follow-up; unresolved food chain considerations	[23,129,130]
Biomaterial-assisted MSC delivery	Multiple large animal species	Experimental scaffold, hydrogel, and encapsulation studies	Improved local retention, survival, mechanotransduction, and tissue integration	Preclinical	Combination product confounding; variable degradation; limited long-term safety or comparative efficacy	[22,105,106,107,116,128,131]
Cell-free MSC products, including EVs and secretome	Dog, horse, and multiple experimental species	In vitro studies, experimental models, and limited early clinical investigations	Transfer of proteins, lipids, and regulatory RNAs; paracrine and immunomodulatory signalling	Investigational	Inconsistent terminology; dose normalization; purity; cargo stability; batch comparability; potency standards	[18,19,56,113,126,127,132,133,134]

* Translational maturity represents a qualitative synthesis based on study design, presence of appropriate controls, independent replication, sample size, naturally occurring versus induced disease, use of objective or structural endpoints, duration of follow-up, safety reporting, and proximity to clinical application. The categories are not formally validated and do not indicate regulatory approval or established standard-of-care status.

**Table 3:** Comparative translational roles of companion and farm animal species in MSC research.

Species	Primary Translational Role	Representative Applications	Major Strengths	Major Limitations	References
Dog	Naturally occurring clinical disease system and veterinary patient	Osteoarthritis; chronic inflammatory enteropathy; atopic dermatitis; spinal cord injury; intervertebral disc repair	Spontaneous chronic disease; clinically relevant pain and functional outcomes; heterogeneous patient populations; strong comparative relevance to human medicine	Breed and disease heterogeneity; variable concurrent treatment; product-specific responses; limited multicentre trials	[20,99,100,101,116,117,121,122,123]
Horse	Naturally occurring high-load musculoskeletal and sports-medicine system	Tendon and ligament injury; joint disease; laminitis; cutaneous wounds; ocular repair	High mechanical loading; structured rehabilitation; longitudinal imaging; return-to-function assessment; reinjury monitoring	Small cohorts; variable athletic discipline and rehabilitation; heterogeneous products; limited controlled structural or mechanical outcomes	[94,95,96,97,102,103,104,108,109,110,113]
Cat	Focused naturally occurring inflammatory-disease system	Chronic gingivostomatitis	Clinically important refractory immune-mediated disease; opportunity to examine treatment sequence and responder heterogeneity	Narrow evidence base; small cohorts; variable response; limited controlled replication	[124,125]
Pig	Human-scale procedural, cardiovascular, and device-oriented model	Myocardial infarction; chronic ischaemic cardiomyopathy; burn repair; catheter-based delivery; biomaterial testing	Clinically relevant organ size; cardiovascular anatomy; advanced imaging; surgery; catheter-based interventions	Predominantly induced disease in young healthy animals; limited chronic comorbidity; incomplete representation of spontaneous disease	[22,111,118,119,120,137]
Sheep	Load-bearing orthopaedic, biomaterial, and reproductive tissue engineering model	Long-bone repair; scaffold evaluation; experimental uterine reconstruction	Clinically relevant skeletal dimensions; fixation systems; implant testing; degradation assessment; mechanical evaluation	Standardized defects often use young healthy animals and may not reproduce chronic degenerative or naturally occurring disease	[48,106,128,138,139]
Goat	Orthopaedic and craniofacial tissue engineering model	Bone regeneration; mandibular reconstruction; scaffold-assisted repair	Surgical accessibility; clinically relevant defect dimensions; evaluation of scaffold integration and mechanical performance	Limited spontaneous disease; induced defects; frequent use of complex combination products	[107,138,139]
Cattle	Production medicine, mammary, and reproductive biological system	Mastitis; uterine inflammation; mammary biology; embryo–maternal communication	Direct agricultural relevance; large animal physiology; milk and reproductive outcomes; herd-level applications	Economic constraints; food chain and biosafety requirements; limited controlled therapeutic trials; difficulty distinguishing inflammatory improvement from bacteriological cure	[23,24,129,130,140,141,142]
Buffalo	Livestock reproductive biology, MSC characterization, and agricultural translation	Culture optimization; hypoxic adaptation; cryopreservation; preliminary treatment of chronic endometritis	Relevance to buffalo reproduction and production systems; opportunity for species-specific manufacturing research	Evidence dominated by laboratory characterization; limited species-specific reagents; preliminary single-centre therapeutic evidence	[25,26,52,143]

The roles shown are complementary rather than hierarchical. Translational value depends on the biological question, disease model, MSC product, administration route, and intended veterinary, agricultural, or comparative outcome.

## Data Availability

No new data were created or analyzed in this study. Data sharing is not applicable to this article.

## Abbreviations

MSCs	Mesenchymal stromal cells
ISCT	International Society for Cellular Therapy
HLA	Human leukocyte antigen
MHC	Major histocompatibility complex
EVs	Extracellular vesicles
MISEV	Minimal Information for Studies of Extracellular Vesicles
IDO1	Indoleamine 2,3-dioxygenase 1
IFN-γ	Interferon-gamma
TNF-α	Tumour necrosis factor-alpha
IL	Interleukin
TLR	Toll-like receptor
PD-1	Programmed cell death protein 1
PD-L1	Programmed death-ligand 1
PD-L2	Programmed death-ligand 2
NK	Natural killer
PGE_2_	Prostaglandin E_2_
TSG-6	Tumour necrosis factor-stimulated gene-6
JAK	Janus kinase
STAT	Signal transducer and activator of transcription
NF-κB	Nuclear factor kappa B
MAPK	Mitogen-activated protein kinase
ERK	Extracellular signal-regulated kinase
PI3K	Phosphoinositide 3-kinase
mTOR	Mechanistic target of rapamycin
HIF-1α	Hypoxia-inducible factor-1 alpha
HIF-TWIST	Hypoxia-inducible factor-1 alpha–TWIST signalling pathway
VEGF	Vascular endothelial growth factor
BMP	Bone morphogenetic protein
TGF-β	Transforming growth factor-beta
YAP	Yes-associated protein
TAZ	Transcriptional co-activator with PDZ-binding motif
OCT4	Octamer-binding transcription factor 4
SOX2	SRY-box transcription factor 2
SSEA	Stage-specific embryonic antigen
